# Reduced-Order Model for Cell Volume Homeostasis: Application to Aqueous Humor Production

**DOI:** 10.3390/mca30010013

**Published:** 2025-01-24

**Authors:** Riccardo Sacco, Greta Chiaravalli, Giovanna Guidoboni, Anita Layton, Gal Antman, Keren Wood Shalem, Alice Verticchio, Brent Siesky, Alon Harris

**Affiliations:** 1 Department of Ophthalmology, Icahn School of Medicine at Mount Sinai, 1468 Madison Avenue, Annenberg 22-86, New York, NY 10029, USA; 2 Dipartimento di Matematica, Politecnico di Milano, Piazza Leonardo da Vinci 32, 20133 Milano, Italy; 3 Dipartimento di Fisica, Politecnico di Milano, Piazza Leonardo da Vinci 32, 20133 Milano, Italy; 4 Maine College of Engineering and Computing, University of Maine, 168 College Avenue, Orono, ME 04469, USA; 5 Department of Applied Mathematics, University of Waterloo, 200 University Ave W, Waterloo, ON N2L 3G1, Canada; 6 Department of Ophthalmology, Rabin Medical Center, Zeev Jabotinsky St 39, Petah Tikva 4941492, Israel; 7 Faculty of Medicine, Tel Aviv University, Klachkin 35 Street, P.O. Box 39040, Tel Aviv 6997801, Israel

**Keywords:** homeostasis, cell volume, homogeneous mixtures, mathematical modeling, aqueous humor production

## Abstract

The ability of a cell to keep its volume constant irrespective of intra- and extracellular conditions is essential for cellular homeostasis and survival. The purpose of this study is to elaborate a theoretical model of cell volume homeostasis and to apply it to a simulation of human aqueous humor (AH) production. The model assumes a cell with a spherical shape and only radial deformation satisfying the property that the cell volume in rest conditions equals that of the cell couplets constituting the ciliary epithelium of the human eye. The cytoplasm is described as a homogeneous mixture containing fluid, ions, and neutral solutes whose evolution is determined by net production mechanisms occurring in the intracellular volume and by water and solute exchange across the membrane. Averaging the balance equations over the cell volume leads to a coupled system of nonlinear ordinary differential equations (ODEs) which are solved using the _*θ*_-method and the Matlab function ode15s. Simulation tests are conducted to characterize the set of parameters corresponding to baseline conditions in AH production. The model is subsequently used to investigate the relative importance of (a) impermeant charged proteins; (b) sodium–potassium (Na+/K+) pumps; (c) carbonic anhydrase (CA) in the AH production process; and (d) intraocular pressure. Results suggest that (a) and (b) play a role; (c) lacks significant weight, at least for low carbon dioxide values; and (d) plays a role for the elevated values of intraocular pressure. Model results describe a higher impact from charged proteins and Na+/K+ ATPase than CA on AH production and cellular volume. The computational virtual laboratory provides a method to further test in vivo experiments and machine learning-based data analysis toward the prevention and cure of ocular diseases such as glaucoma.

## 1. Introduction

The mechanical integrity of a cell is essential to its function and the tissues and organ systems that it supports [1,2]. Cell volume stabilization is a fundamental function to preserve the mechanical integrity of a cell [3]. Cell volume stabilization over time is based on the balance among forces acting on the cell membrane from both intra- and extracellular sides, which emanate from electrochemical and fluid–mechanical mechanisms [4]. The experimental characterization of force equilibrium at the nanoscale level is a highly complex task, on the one hand, because of the difficulty of performing measurements in vivo (see [5]) and, on the other hand, the presence of two regimes of mechanical behavior in living cells, on short and long time scales (see [6]). In fact, a combination of experimentation and theoretical analysis shows that living mammalian cytoplasm behaves as an equilibrium material on short time scales whereas it behaves as an out-of-equilibrium material on long time scales [6].

Based on these considerations, in this article we address the theoretical study of cell volume dynamics and apply the formulation to the process of the production of aqueous humor (AH) in the eye. AH is a slowly moving fluid that is continuously produced by the ciliary body of the eye, whose main function is to keep the anterior segment of the eye clean from metabolic wastes and to preserve the spherical shape of the eyeball by establishing the intraocular pressure (IOP) (see [7–10]).

The clinical motivation of our work is the fact that an elevated value of intraocular pressure (IOP) is an established risk factor for glaucoma. Therefore, keeping IOP within normal levels (10 to 21 mmHg) is central to prevent the occurrence and progression of neurodegenerative diseases of the retina such as glaucoma [11–13]. IOP is the result of the balance between the production and drainage of aqueous humor (AH) in the anterior segment of the eye. AH is produced by the ciliary epithelium (CE) double cell layer, which lines the ciliary processes of the ciliary body, and is drained out from the anterior chamber of the eye throughout the trabecular meshwork and uveoscleral outflow pathways. In this article, we focus our attention on the process of AH production with the goal of characterizing the fluid–mechanical and electrochemical conditions under which the cells constituting the CE maintain their volume, allowing at the same time stationary water flow from the ciliary body toward the posterior and anterior chamber of the eye. Understanding these conditions endows clinicians with a supporting tool to identify the causes of a pathological increase (or decrease) in AH production which may give rise to a potentially pathological increase in IOP, the final aim being to devise effective therapies for its reduction [14]. In this respect, the development of a mathematical model and of a computational virtual laboratory (CVL) for the simulation of AH production has been the object of investigation in recent years (see [15–18]).

In this article, we propose a theoretical formulation based on homogeneous mixtures including neutral and charged solutes (see [19] (Chapter 13)) and utilizing a model reduction procedure from three spatial dimensions to zero spatial dimensions. The model resulting from the reduction is a coupled system of highly stiff, nonlinear, ordinary differential equations (ODEs) which are solved using the *θ*-method and the Matlab function ode15s. Simulation tests are run to characterize the values of model parameters in baseline conditions in AH production. The model is subsequently used to investigate the relative importance of (a) impermeant charged proteins; (b) the sodium–potassium pump; (c) carbonic anhydrase; and (d) intraocular pressure in the AH production process. Results suggest that (a) and (b) play a role; (c) does not have a significant weight, at least for low CO_2_ values; and (d) plays a role for elevated values of intraocular pressure, as in the case of hypertensive patients.

## 2. Materials and Methods

In Section 2.1, we describe the cellular mechanisms of AH production. In Section 2.2, we describe the geometrical representation of the cell volume and surface; in Section 2.3, we describe the heterogeneous structure of the cell membrane; and in Section 2.4, we provide a conceptual scheme of the transport of water and solute across the cell membrane. In Section 2.5, we introduce a continuum model of the cell based on the theory of mixtures to describe the spatial and temporal response of the cell volume to the concomitant action of fluid and electrochemical forces. In Section 2.6, we derive a reduced-order model of the cell volume that comprises ordinary differential and algebraic equations. Finally, in Sections 2.7 and 2.8, we describe the compact form of the reduced-order model and its numerical approximation, respectively. In Appendices A and B, we provide the expressions of the fluid velocity, molar flux densities, and net production rates that are involved in cellular metabolism.

### 2.1. Cellular Mechanisms of Aqueous Humor Production

**Figure 1.**
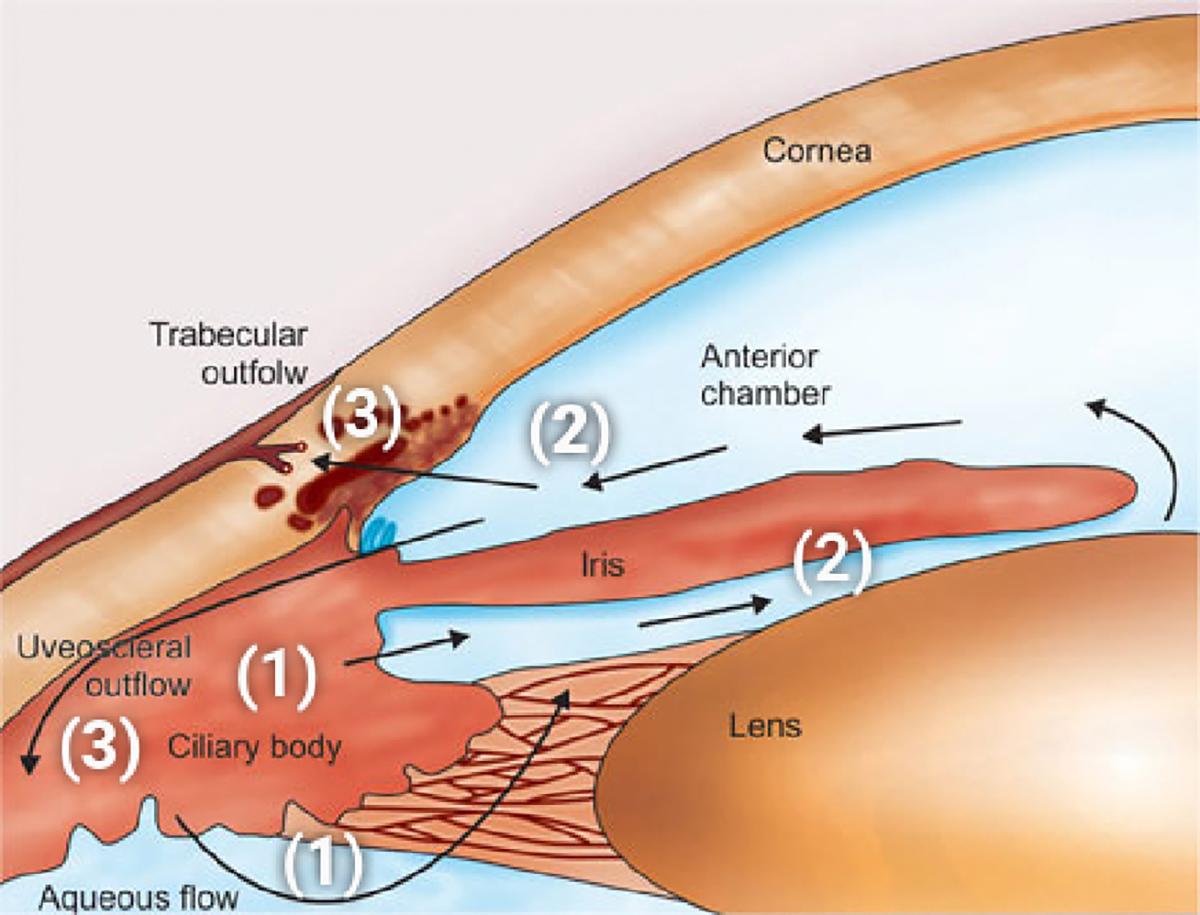
A schematic representation of the processes involved in AH dynamics. (1): AH production; (2): AH flow; (3): AH outflow. Reprinted from R. Ramakrishnan et al, *Diagnosis & Management of Glaucoma*, Chapter 9 Aqueous Humor Dynamics, 10.5005/jp/books/11801_9, (2013) [21]; used in accordance with the Creative Commons Attribution (CC BY) license.

Aqueous humor (AH) is a slowly moving fluid that is (1) continuously produced by the ciliary body epithelium of the eye (CE); (2) flows from the posterior to the anterior chamber of the eye; and (3) is drained out from the eye throughout two main outflow pathways (see [7–10]). The sequence (1) AH production, (2) AH flow, and (3) AH outflow is schematically represented in Figure 1. The main function of AH is to keep the anterior segment of the eye clean from the metabolic waste of the surrounding tissues and to preserve the spherical shape of the eyeball by establishing the intraocular pressure (IOP) as the value of the AH fluid pressure. This corresponds to the volumetric flow rate of AH that is produced by the ciliary body and equals the volumetric flow rate of AH that is drained out by the outflow pathways in the anterior segment of the eye (see [20]).

**Figure 2.**
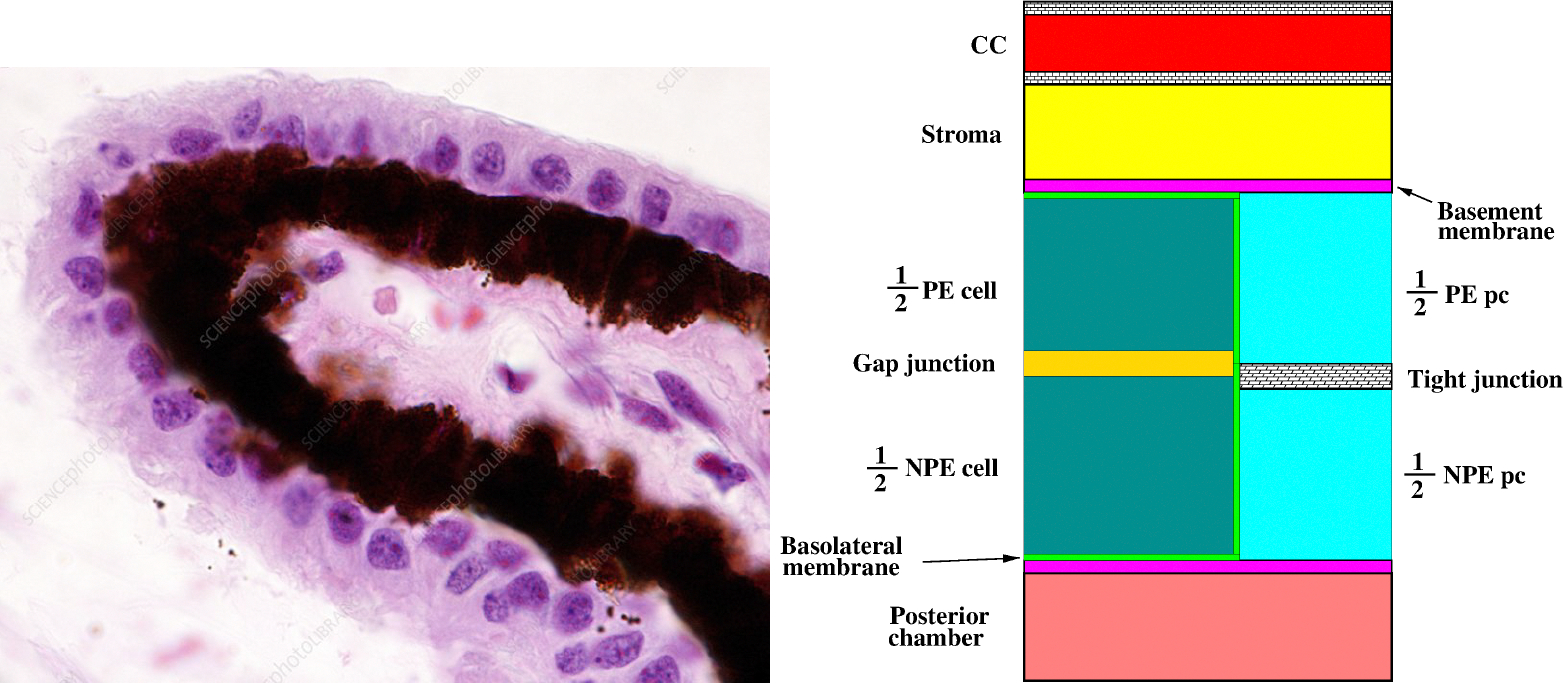
**Left** panel: a light micrograph of the ciliary body epithelium. It consists of two epithelial cell layers: a non-pigmented inner layer and an outer pigmented layer. Under the epithelium, there is a highly vascularized stroma. Reprinted from [23]; used in accordance with the Creative Commons Attribution (CC BY) license. **Right** panel: a compartmental representation of the CE cell couplet.

The left panel of Figure 2 is a micrograph photo of a histology of the CE. The CE is a double layer of apex-to-apex connected cells (pigmented cell, PE, and nonpigmented cell, NPE) which have cuboidal and columnar shapes, respectively (see [22]). The CE cell couplet is schematically illustrated in the compartment-based representation shown in the right panel of Figure 2. This scheme may be used to characterize the various cellular mechanisms that concur in the process of AH production (see [9,20]). The first mechanism is the transport of the “metabolic fuel”, a mixture constituted by water, solutes, ions, and proteins, by blood flow in the ciliary capillary (CC). The second mechanism is the ultrafiltration of the metabolic fuel across the fenestrations in the CC wall. The third mechanism is the motion of the metabolic fuel across the stromal tissue. Then, the metabolic fuel divides across two different pathways. The first pathway is the intracellular transport throughout the CE cell couplet; the second pathway is the paracellular transport throughout the lateral interstitial space separating two neighbouring CE cell couplets. In its motion across the CE, the osmolarity gradient that is established between the cytoplasm of the NPE cell and the interstitial space, on the one hand, and between the NPE cell and the posterior chamber (PC), on the other hand, gives rise to a water efflux into the PC which eventually drains out through the trabecular meshwork and uveoscleral outflow pathways.

### 2.2. Geometrical Description of the Cell

**Figure 3.**
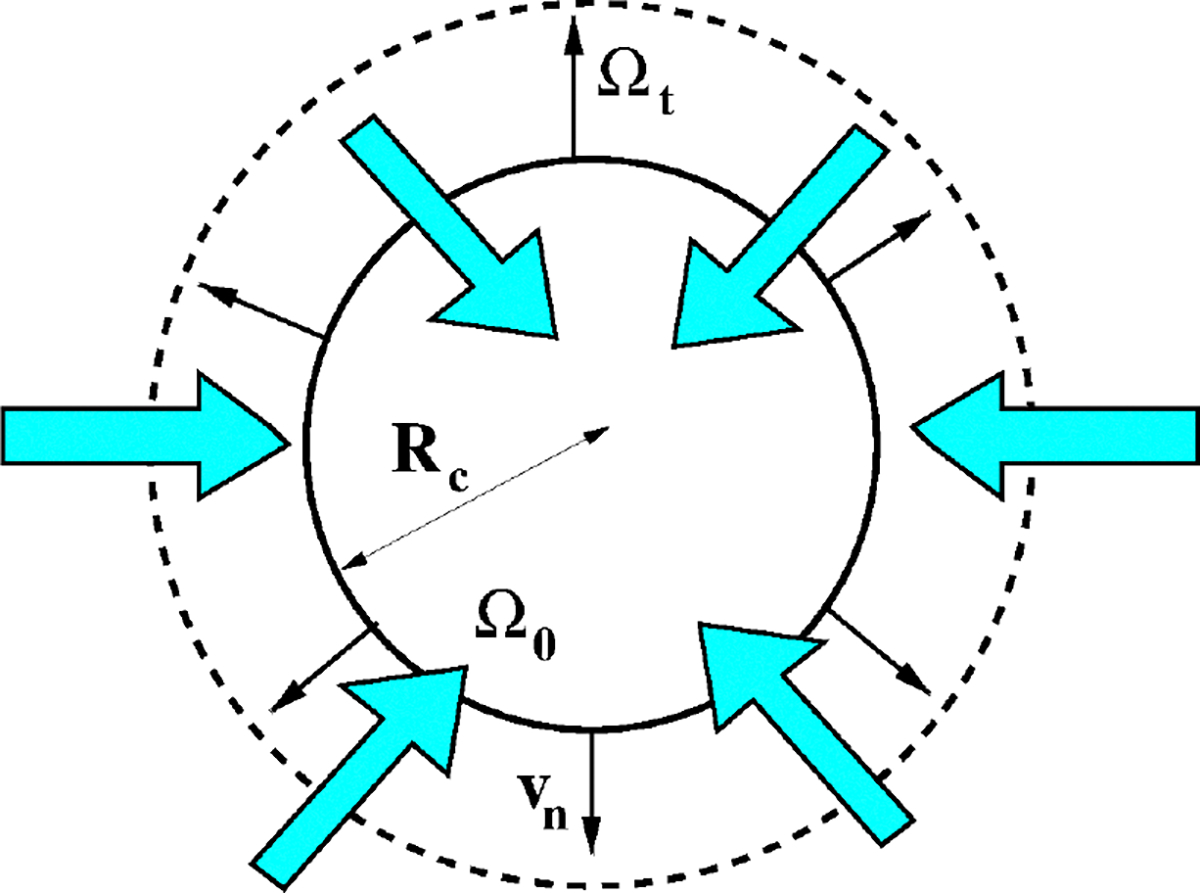
The “equivalent cell”. Solid line: initial cell configuration. Dashed line: deformed cell configuration. The cyan arrows indicate water flow. The cell is increasing its volume (cell swelling).

Figure 3 illustrates the “equivalent cell”, representing a simplified model of the CE cell couplet illustrated in Section 2.1. The “equivalent cell” has the property that its volume is equal to the volume of the PE/NPE cell couplet, and the reason supporting the choice of a spherical shape is that the PE/NPE cell couplet acts as a functional syncytium [8] so that the process of AH production can be mathematically described as the collective contribution of all the cell couplets that constitute the CE. Despite its over-simplified geometry, we believe that our model may serve as a basis for extension to future closer representations of the morphology and physiology of the CE.

Let t≥0 denote the time variable (units: s). We denote by $\Omega_{t}:=\Omega (t)$ the three-dimensional (3D) body representing the cell at the time t and ${𝒱}_{t}:=𝒱(t)$, the volume of the cell at the time t, such that ${𝒱}_{0}=4\pi R_{c}^{3}/3$ is the value of the cell volume at the time t=0, with $R_{c}$ being the cell radius in the initial (undeformed) configuration (units: m). From the point of view of Continuum Mechanics, the body is a deformable, electrically charged, homogeneous mixture comprising a fluid constituent, with $N_{\alpha }$ moving charged solute constituents (ions) and $N_{\beta }$ moving neutral solute constituents (see [19] (Chapter 13)). The cell volume also contains impermeant charged proteins whose chemical valency and molar density are such that electroneutrality holds in ionic homeostasis conditions (see [3,24]). The boundary of $\Omega_{t}$ is denoted by $\partial \Omega_{t}$ and n is the outward unit normal vector for $\partial \Omega_{t}$. The surface of the cell at the time t is denoted by ${𝒮}_{t}:=𝒮(t)$, such that ${𝒮}_{0}=4\pi R_{c}^{2}$ is the value of the cell surface at the time t=0. In the remainder of this article, we denote by x the spatial coordinate vector of any point in the cell with respect to a fixed system of reference. We also refer to the interior of $\Omega_{t}$ as the intracellular region (shortly, *in*), to the exterior of $\Omega_{t}$ as the extracellular region (shortly, *out*), and to the surface of $\Omega_{t}$ as the membrane (shortly, m ).

#### Assumption 1.

We assume that cell deformation occurs only in the radial direction and that it does not depend on the position on the cell surface.

#### Assumption 2.

We assume that the electric potential, solute concentrations, and fluid pressure in the extracellular region are given functions of space and time.

The solid line and dashed line in Figure 3 illustrate the initial and deformed configurations of the cell, respectively. In agreement with Assumption 1, both configurations are spherical.

### 2.3. Porous and Lipid Structure of Cell Membrane

The intra- and extracellular regions are separated by a 3D membrane whose thickness, $t_{m}$, is such that the ratio $\delta :=t_{m}/R_{c}$ is ≪1. In the limit δ→0, the geometrical representation of the 3D membrane degenerates into the two-dimensional (2D) manifold $\partial \Omega_{t}$. The membrane surface can be represented as a heterogeneous porous medium whose solid constituent is lipid material. The lipid surface is impermeable to water and solutes, whereas the pore surface allows the exchange of fluid and solutes between intra- and extracellular regions. The pore surface contains a variety of membrane proteins, denoted by mp. Each membrane protein, mp, is characterized by a dimensionless function, $\Phi^{mp}$, henceforth referred to as the surface fraction, defined as

(1a) $$\Phi^{mp}(t):=\frac{{𝒮}^{mp}(t)}{𝒮(t)}\;𝒮(t)>0,\;t\geq 0,$$

where ${𝒮}^{mp}(t)$ is the total area occupied by the protein mp at the time t.

#### Assumption 3.

*Let* ${𝒮}_{t}>0$. *We assume that*

(1b) $$\frac{d\Phi^{mp}(t)}{dt}=0\;t>0.$$

Equation (1b) *implies that*

(1c) $$\Phi^{mp}(t)=\Phi^{mp}(0)=\frac{{𝒮}^{mp}(0)}{𝒮(0)}\;t\geq 0.$$

The first type of membrane protein that we introduce in this article is an aquaporin (AQP), which is a specialized protein for rapid transmembrane water exchange between intracellular and extracellular regions (see [25–27]). The quantity $\Phi^{AQP}$ represents the AQP surface fraction. Other membrane proteins on the cell surface comprise carrier proteins, which permit neutral solute exchange through the cell membrane by the mechanism of facilitated diffusion (see [28,29]), and ion channels, ion exchangers, and ion pumps, which permit charged solute exchange through the cell membrane (see [30]). The total surface fractions for each considered membrane protein are defined as

(2a) $$\Phi^{carr}=\sum_{\beta \in S_{\beta }}\Phi_{\beta }^{carr},$$

(2b) $$\Phi^{ch}=\sum_{\alpha \in S_{\alpha }}\Phi_{\alpha }^{ch},$$

(2c) $$\Phi^{\mathrm{exch}}=\sum_{\alpha \in S_{\alpha }}\Phi_{\alpha }^{\mathrm{exch}},$$

(2d) $$\Phi^{\mathrm{pump}}=\sum_{\alpha \in S_{\alpha }}\Phi_{\alpha }^{\mathrm{pump}},$$

in such a way that the total pore surface fraction is

(3) $$\Phi_{\mathrm{tot}\;}^{p}=\Phi^{\mathrm{carr}\;}+\Phi^{\mathrm{ch}\;}+\Phi^{\mathrm{exch}\;}+\Phi^{\mathrm{pump}\;}+\Phi^{AQP}.$$

The remainder of the cell surface is occupied by the lipid constituent of the membrane, whose surface fraction is

(4) $$\Phi^{lip}=1-\Phi_{tot}^{p}.$$

Assumption 3 implies that the membrane protein surface fractions in (2) do not depend on the time and also that the lipid surface fraction does not depend on the time because of (4).

### 2.4. Water and Solute Transport Across the Cell Membrane

In this section, we provide a conceptual scheme of the transport of water and solute across the cell membrane and we refer to [26,31] for more details about the subject.

**Figure 4.**
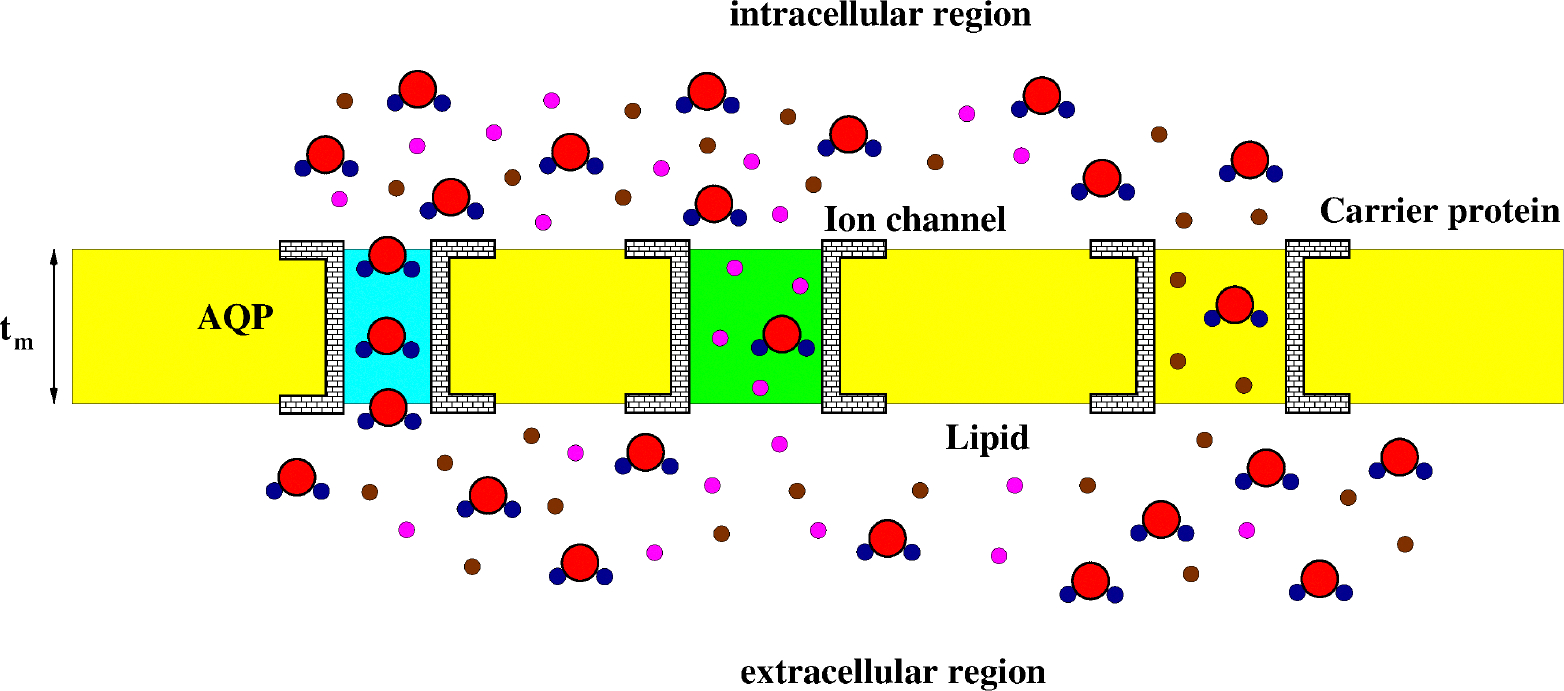
A schematic representation of the structure of the cell membrane. AQP: aquaporin (cyan). The ion channel is drawn in green. Water molecules (red and dark blue), charged solutes (magenta), and neutral solutes (brown) are illustrated. The lipid constituent is drawn in yellow. The AQP is selective to water molecules whereas the ion channel permits the co-transport of ions and water.

Figure 4 illustrates a schematic representation of a zoomed view of the cell membrane and of the intra- and extracellular regions. The scheme shows water molecules and solutes (neutral and charged) in motion across the membrane.

#### Assumption 4.

*Based on the scheme in* Figure 4, *we make the following assumptions*:

**A1.** *Water is transported across the membrane, in a selective manner, via aquaporins;*

**A2.** *Water and solutes are co-transported across the membrane via ionic channels and carrier proteins;*

**A3.** *Water velocity inside ionic channels and carrier proteins is the same as water velocity inside aquaporins.*

### 2.5. Continuum-Based Model of Cell

This section is structured with four subsections devoted to fluid motion, neutral and charged solutes, and electric potential.

#### 2.5.1. Fluid Motion

##### Assumption 5.

*We assume that water flow is slow and inertial forces can be neglected with respect to viscous effects.*

According to Assumption 5, the motion of water flow across the cell membrane can be described by the linear Stokes equation system (see [19] (Section 10.5.1)):

(5a) ∇⋅v=0,

(5b) ∇⋅T+b=0,

(5c) $$T=-pI+2\mu_{f}\nabla_{S}v,$$

where v (units: ms^−1^) and p (units: Nm^−2^) are the fluid velocity and pressure inside the aquaporin, respectively, T is the fluid stress tensor (units: Nm^−2^), b is the force density acting on the fluid (units: Nm^−3^), $\mu_{f}$ is the fluid dynamic viscosity (units: Nm^−2^ s = Pa s), and $\nabla_{S}$ is the symmetric gradient operator. Equation (5a) expresses mass conservation in a local form; Equation (5b) expresses linear momentum balance in a local form; and Equation (5c) is the constitutive law for a Newtonian fluid.

#### 2.5.2. Neutral Solutes’ Motion

We denote by $S_{\beta }$ the set of neutral solutes, with $card\left(S_{\beta }\right)=N_{\beta }$. Each solute, $\beta \in S_{\beta }$, is described by its number density, $n_{\beta }$, and molar density, $c_{\beta }$ (units: mol m^−3^= mM). The advection–diffusion model is adopted to represent the motion of neutral solutes across the cell membrane (see [19] (Chapter 12)):

(6a) $$\frac{\partial c_{\beta }}{\partial t}+\nabla \cdot j_{\beta }=P_{\beta }-C_{\beta }\;\beta =1,\ldots ,N_{\beta },$$

(6b) $$j_{\beta }=c_{\beta }v-D_{\beta }\nabla c_{\beta }\;\beta =1,\ldots ,N_{\beta }.$$

Equation (6a) expresses mass balance in a local form for each solute, $\beta \in S_{\beta }$. The vector $j_{\beta }$ is the molar flux density of the solute β (units: mol m^−2^ s^−1^= mM ms^−1^), accounting for a passive advective contribution due to water motion inside the carrier protein channel (see Assumption 4-A2) and a diffusive contribution expressed by Fick’s law in which $D_{\beta }$ is the diffusion coefficient of the solute β (units: m^2^ s^−1^). The scalar functions $P_{\beta }$ and $C_{\beta }$ represent the production and consumption rates of $c_{\beta }$ (units: mol m^−3^ s^−1^= mMs^−1^).

#### 2.5.3. Charged Solutes’ Motion

We denote by $S_{\alpha }$ the set of charged solutes (ions) with card $\left(S_{\alpha }\right)=N_{\alpha }$. Each ion, $\alpha \in S_{\alpha }$, is described by its number density, $n_{\alpha }$, molar density, $c_{\alpha }$, charge number, $z_{\alpha }$, diffusion coefficient, $D_{\alpha }$, and electric mobility, $\mu_{\alpha }^{el}$ (units: m^2^ V^−1^ s^−1^). The number and molar densities are related by the equation $n_{\alpha }=N_{Av}c_{\alpha }$ (units: m^−3^), where $N_{Av}=6.02214076\cdot \;10^{23}{\;mol}^{-1}$ is Avogadro’s constant. The diffusion coefficient and the electric mobility are proportional through Einstein’s relation:

(7) $$D_{\alpha }=\frac{\mu_{\alpha }^{el}V_{th}}{\left|z_{\alpha }\right|}\;\alpha =1,\ldots ,N_{\alpha },$$

where $V_{th}=\left(K_{B}T\right)/q$ is the thermal voltage (units: V ) and $K_{B}$, T, and q denote Boltzmann’s constant, absolute temperature, and electron charge, respectively. The Nernst–Planck (NP) model is adopted to represent ion transport and exchange across the cell membrane (see [19] (Chapter 13)):

(8a) $$\frac{\partial c_{\alpha }}{\partial t}+\nabla \cdot j_{\alpha }=P_{\alpha }-C_{\alpha }\;\alpha =1,\ldots ,N_{\alpha },$$

(8b) $$j_{\alpha }=j_{\alpha }^{p}+j_{\alpha }^{a}\;\alpha =1,\ldots ,N_{\alpha },$$

(8c) $$j_{\alpha }^{p}=j_{\alpha }^{p,edw}+j_{\alpha }^{p,exch\;}\;\alpha =1,\ldots ,N_{\alpha },$$

(8d) $$j_{\alpha }^{p,edw\;}=c_{\alpha }v_{\alpha }-D_{\alpha }\nabla c_{\alpha }\;\alpha =1,\ldots ,N_{\alpha },$$

(8e) $$v_{\alpha }=v+\mu_{\alpha }^{el}\frac{\left|z_{\alpha }\right|}{z_{\alpha }}E.$$

Equation (8a) expresses mass balance in a local form for each ion $\alpha \in S_{\alpha }$. Equation (8b) is the constitutive law to describe ion transport and contains two main contributions to the vector $j_{\alpha }$ which is the molar flux density of the ion α. The first contribution accounts for passive ion transport and is represented by the ion molar flux density $j_{\alpha }^{p}$. The second contribution, which is a novel aspect introduced by the model proposed in the present work, accounts for active ion transport and is represented by the ion molar flux density $j_{\alpha }^{a}$. The vector $j_{\alpha }^{p,edw}$ is the velocity-extended NP equation for ion electrodiffusion and accounts for a passive advective contribution and a diffusive contribution expressed by Fick’s law (see [19] (Chapter 13)). The vector field $v_{\alpha }$ is the generalized drift velocity of the ion α due to water motion inside the ion channel (see Assumption 4-A2) and Faraday’s law under quasi-static approximation (see [19] (Chapter 14))

(8f) E=-∇ψ,

where E and ψ are the electric field (units: Vm^−1^) and electric potential (units: V), respectively. The vector $j_{\alpha }^{p,exch}$ expresses passive ion exchange mediated by transporters and co-transporters. The scalar functions $P_{\alpha }$ and $C_{\alpha }$ represent the production and consumption rates of $c_{\alpha }$.

#### 2.5.4. Electric Potential

In the classical VE-PNP equation system, the Poisson equation,

(9) $$\nabla \cdot \left(-\varepsilon_{m}\nabla \psi \right)=\rho^{el},$$

is used to determine the spatial distribution of the electric potential, with $\varepsilon_{m}$ denoting the dielectric permittivity of the medium (units: F m^−1^) and $\rho^{el}$ denoting the electric charge density (units: Cm^−3^). Since, in the present article, we are interested in the study of the spatial and temporal distribution of the electric potential ψ across the cell membrane, we replace (9) (which, for a given $\rho^{el}$, is an elliptic equation) with the following differential system:

(10a) $$\nabla \cdot J_{tot}=0,$$

(10b) $$J_{tot\;}=J^{disp\;}+J_{tot\;}^{cond\;},$$

(10c) $$J^{disp}=\frac{\partial }{\partial t}\left(\varepsilon_{m}E\right)=\frac{\partial }{\partial t}\left(-\varepsilon_{m}\nabla \psi \right),$$

(10d) $$J_{tot\;}^{cond}=F\sum_{\alpha \in S_{\alpha }}z_{\alpha }j_{\alpha },$$

where F is Faraday’s constant (units: Cmol^−1^). Equation (10a) is a consequence of Maxwell’s equations (see [19,32] (Chapter 4)) and expresses the continuity of the total electric current density $J_{tot}$ (units: Cm^−2^ s^−1^= Am^−2^), given by the sum of the total conduction current density $J_{tot\;}^{cond\;}$ and the displacement current density $J^{disp\;}$, in which the electric field E is expressed as a function of the electric potential ψ via Equation (8f). The adoption of System (10) to determine the electric potential ψ is a novel aspect of this article and extends to the time-dependent case of the approach originally proposed in [33] to enforce electroneutrality in the mathematical study of cellular electric activity.

### 2.6. Reduced-Order Cell Model

This section is structured with five subsections devoted to the derivation of the reduced-order model for the cell surface normal velocity, cell volume, neutral and charged solute molar densities, and electric potential.

#### 2.6.1. Time Evolution of Normal Velocity Across Single AQP

We make the following assumption about the geometry of the AQP shown in Figure 4.

##### Assumption 6.

**Figure 5.**
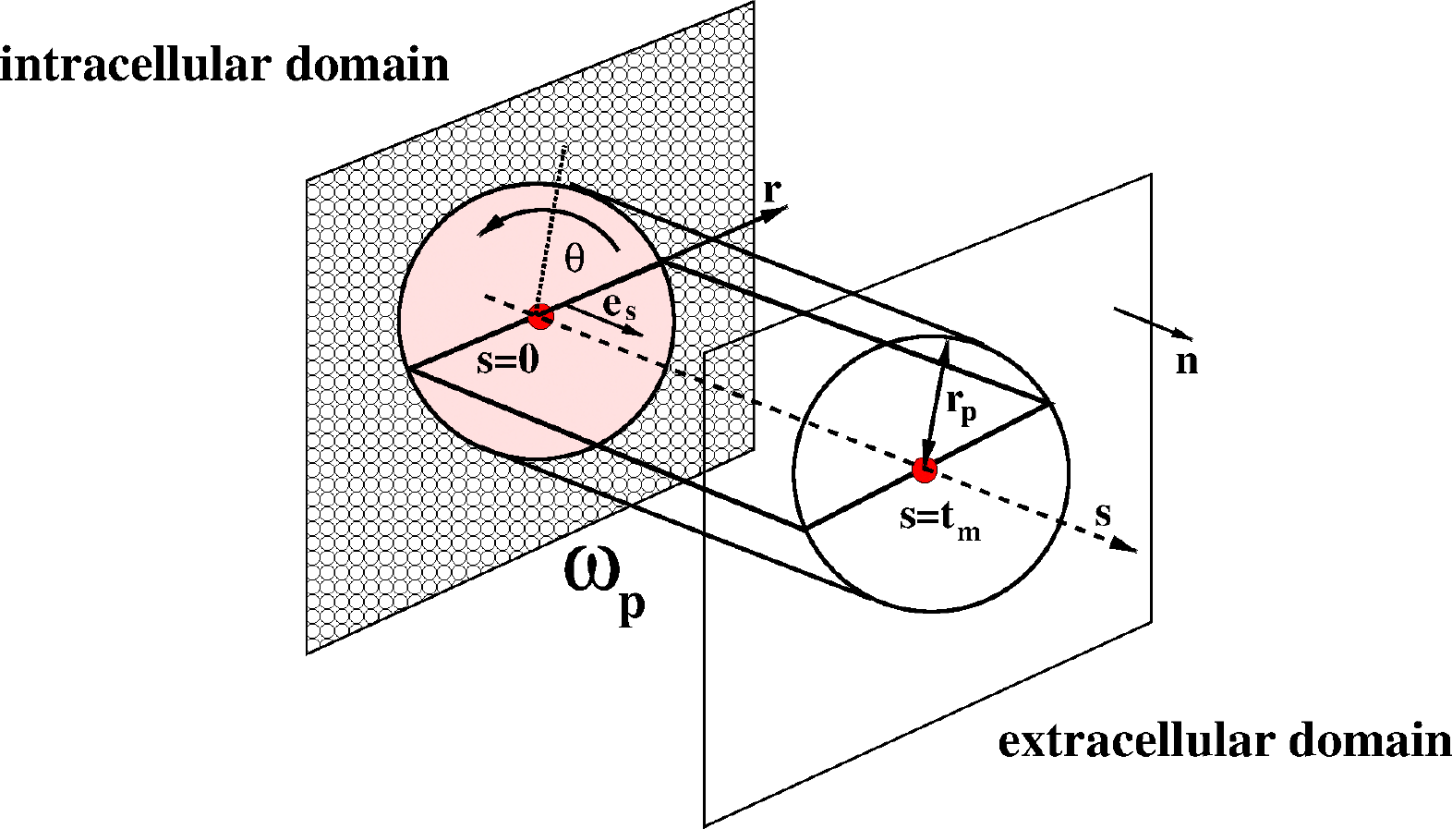
A three-dimensional schematic representation of an aquaporin. The cylindrical domain $\omega_{p}$ is the pore channel, $t_{m}$ is the membrane thickness, and $r_{p}$ is the aquaporin radius.

*The* AQP *is geometrically represented as a cylinder*, $\omega_{p}$, *with radius* $r_{p}$ *and axial length* $t_{m}$ *, as depicted in* Figure 5.

Moreover, we make the following assumption:

##### Assumption 7.

We assume the following:

1. *The fluid velocity only has the axial component* $V_{s}$;
2. $V_{s}=v_{p,n}(t)\eta (r)$, *with* t≥0 *and* $r\in \left[0,r_{p}\right]$;
3. $\eta (r)=2\left[1-{\left(r/r_{p}\right)}^{2}\right]$ *(Poiseuille flow)*;
4. *The force density only has the axial component* $b_{s}$;
5. $b_{s}=b_{p,s}(s,t)$, *with* $s\in \left[0,t_{m}\right]$ *and* t≥0.

Inserting Assumption 7 into Equation (5), we obtain the following expression of the normal fluid velocity inside the pore channel of the AQP:

(11a) $$v_{p,n}(t)=\frac{r_{p}^{2}}{8\mu_{f}}\left[-\frac{\partial p(s,\;t)}{\partial s}+b_{p,s}(s,\;t)\right]\;s\in \left[0,t_{m}\right],t\geq 0.$$

Integrating (11a), we obtain

(11b) $$p(s,t)=p(0,t)-\frac{8\mu_{f}v_{p,n}(t)}{r_{p}^{2}}s+\int_{0}^{s}b_{p,s}(\xi ,t)d\xi \;s\in \left[0,t_{m}\right],t\geq 0,$$

For any function, U=U(t), we define the transmembrane difference operator:

(11c) $$\Delta U(t):=U_{in\;}(t)-U_{out\;}(t)\;t\geq 0.$$

Setting $s=t_{m}$ in (11b) and applying the definition (11c) to the variables p and Π, we obtain

(11d) $$v_{p,n}(t)={ℒ}_{p}[\Delta p(t)-\Delta \Pi (t)]\;t\geq 0,$$

Where

(11e) $${ℒ}_{p}:=\frac{r_{p}^{2}}{8\mu_{f}t_{m}}$$

is the hydraulic conductivity of the membrane (units: m(Pa s)^−1^) and

(11f) $$\Pi (t):=\int b_{p,s}(\xi ,t)d\xi +C$$

is the total osmo-oncotic pressure, with C being an arbitrary integration constant. Relation (11d) is Starling’s equation [34] and represents the mathematical expression of the normal fluid velocity across a single aquaporin.

#### 2.6.2. Time Evolution of Cell Surface Normal Velocity

In order to determine the motion of the whole cell surface, we need to define an average normal fluid velocity to represent the collective contribution of the AQPs. For this purpose, we denote by $\sigma_{AQP}$ the surface density of aquaporins, defined as the number, $N_{AQP}$, of AQPs per square meter, and by ${𝒮}_{AQP}^{tot\;}(t)$ the total surface area occupied by the AQPs. Using Assumption 3, we obtain

(12a) $$\Phi^{AQP}=\frac{{𝒮}_{AQP}^{tot\;}(0)}{{𝒮}_{0}}=\frac{\sigma_{AQP}{𝒮}_{0}\pi r_{p}^{2}}{{𝒮}_{0}}=\sigma_{AQP}\pi r_{p}^{2}.$$

Let us introduce the total water volumetric flow rate across the cell surface (units: m^3^ s^−1^),

(12b) $$Q_{w}(t):=\int_{\partial \Omega_{t}}v(x,t)\cdot nd\left(\partial \Omega_{t}\right)=v_{p,n}(t){𝒮}_{AQP}^{tot\;}(t)\;t\geq 0,$$

and the average normal fluid velocity across the cell surface,

(12c) $$\langle v(x,t)\cdot n\rangle (t):=\frac{Q_{w}(t)}{{𝒮}_{t}}=\frac{{𝒮}_{AQP}^{tot}(t)}{{𝒮}_{t}}v_{p,n}(t)=\Phi^{AQP}v_{p,n}(t)\;t\geq 0,$$

where $\Phi^{AQP}$ is given by (12a) and $v_{p,n}$ is given by (11d).

The pore fluid velocity $v_{p,n}$ is typically quite large (even thousands of μm s^−1^; see [35]) to favor a fast transmembrane exchange of water molecules. The average normal fluid velocity ⟨v⋅n⟩, instead, is considerably smaller (less that 1 μm s^−1^; see [36]). For this reason, in the remainder of this article, we introduce the following definition.

##### Definition 1.

*Let us denote by* $v_{cell,n\;}$ *the cell surface normal velocity. We set*

(13) $$v_{cell,n}(t):=\langle v(x,t)\cdot n\rangle =\Phi^{AQP}v_{p,n}(t)\;x\in \partial \Omega_{t},t\geq 0,$$

*where* $v_{p,n}$ *is defined in* (11d).

#### 2.6.3. Time Evolution of Cell Volume

The time evolution of ${𝒱}_{t}$ is described by the cell mass balance equation in an integral form (see [19] (Chapter 6)):

(14) $$\frac{dM(t)}{dt}=-\int_{\partial \Omega_{t}}\rho_{w}v(x,t)\cdot nd\left(\partial \Omega_{t}\right)+\int_{\Omega_{t}}R_{w}(x,t)d\Omega_{t}\;t\geq 0,$$

where $\rho_{w}$ is the mass density of water (units: kg m^−3^), $M(t)=\int_{\Omega_{t}}\rho_{w}d\Omega_{t}$ is the mass of the cell at the time t (units: kg), and

(15) $$R_{w}\left(x,t\right)≔P_{w}\left(x,t\right)-C_{w}\left(x,t\right)\;x\in \Omega_{t},t\geq 0$$

is the intracellular water mass density net production rate, with $P_{w}$ and $C_{w}$ being the water mass density production and consumption rates, respectively (units: kg m^−3^ s^−1^).

##### Assumption 8.

*We assume that*

(16a) $$P_{w}(x,t)={𝒫}_{w}(t)\Phi (x)\;x\in \Omega_{t},t\geq 0,$$

(16b) $$C_{w}(x,t)={𝒞}_{w}(t)\Phi (x)\;x\in \Omega_{t},t\geq 0,$$

*where* ${𝒫}_{w}$ *and* ${𝒞}_{w}$ *represent the time-dependent intracellular water mass density production and consumption rates, and the shape function* Φ *is such that*

(16c) $$\int_{\Omega_{t}}\Phi \left(x\right)\;d\Omega_{t}={𝒱}_{t}.$$

Using Assumption 4-A1, using Definitions 1 and 8 in the right-hand side of (14), and noting that $𝒮(t)=\gamma (𝒱(t){)}^{2/3}$, with $\gamma :=(36\pi {)}^{1/3}$, we obtain the following reduced-order model of cell volume motion:

(17a) $$\frac{d𝒱(t)}{dt}=-\gamma j_{v,n}(t)(𝒱(t){)}^{2/3}+{ℛ}_{w}(t)𝒱(t)\;t>0,$$

(17b) $$j_{v,n}(t):=v_{cell,n}(t)=\Phi^{AQP}v_{p,n}(t)\;t\;>0,$$

(17c) $$𝒱(0)={𝒱}_{0},$$

where $v_{p,n}$ is given by (11d) and

(17d) $${ℛ}_{w}\left(t\right)≔k_{w,prod}\left(t\right)-k_{w,cons}\left(t\right)\;t\geq 0$$

is the water volume net production rate (units: s^−1^), with $k_{w,prod}(t):={𝒫}_{w}(t)/\rho_{w}$ and $k_{w,cons\;}(t):={𝒞}_{w}(t)/\rho_{w}$ set for every t≥0.

#### 2.6.4. Time Evolution of Neutral Solutes

The time evolution of neutral solute molar density is described by the mass balance equation in an integral form:

(18) $$\int_{\Omega_{t}}\frac{\partial c_{\beta }}{\partial t}d\Omega_{t}=-\int_{\partial \Omega_{t}}j_{\beta ,n}d\left(\partial \Omega_{t}\right)+\int_{\Omega_{t}}R_{\beta }d\Omega_{t}\;\beta \in S_{\beta },$$

where $j_{\beta ,n}$ is the normal molar flux density of $c_{\beta }$ over the cell surface ${𝒮}_{t}$, and

(19) $$R_{\beta }\left(x,t\right)≔P_{\beta }\left(x,t\right)-C_{\beta }\left(x,t\right)\;x\in \Omega_{t},\;t\geq 0$$

is the net production rate of the intracellular solute β, with $P_{\beta }$ and $C_{\beta }$ being the production and consumption rates, respectively (units: mMs^−1^).

##### Assumption 9.

*We assume that*

(20a) $$c_{\beta }\left(x,t\right)=c^{\beta }\left(t\right)\Phi \left(x\right)\;x\in \Omega_{t},\;t\geq 0,$$

(20b) $$j_{\beta ,n}(x,t)=j_{n}^{\beta }(t)\eta_{\beta }(x)\;x\in \partial \Omega_{t},\;t\geq 0,$$

*where* $c^{\beta }$ *and* $j_{n}^{\beta }$ *are time-dependent intracellular neutral solute molar densities and normal molar flux densities on the cell surface, respectively, and the shape function* $\eta_{\beta }$ *is such that*

(20c) $$\int_{\partial \Omega_{t}}\eta_{\beta }\left(x\right)\;d\left(\partial \Omega_{t}\right)=\Phi_{\beta }^{carr\;}{𝒮}_{t},$$

*where* $\Phi_{\beta }^{carr\;}$ *is the surface fraction of the carrier protein that allows the transmembrane exchange of the neutral solute* β. *We also assume that equations similar to* (16) *apply to* $P_{\beta }\;and\;C_{\beta }$.

Using Assumption 9 in (18), we obtain the following reduced-order model for each neutral solute molar density:

(21a) $$\frac{dc^{\beta }(t)}{dt}=-\gamma j_{\beta ,n}^{tot}(t)(𝒱(t){)}^{-1/3}+{ℛ}_{\beta }(t)\;\beta \in S_{\beta },t>0,$$

(21b) $$j_{\beta ,n}^{tot}(t):=\Phi_{\beta }^{carr}j_{n}^{\beta }(t)\;\beta \in S_{\beta },t>0,$$

(21c) $$c^{\beta }\left(0\right)=c_{0}^{\beta },$$

where $c_{0}^{\beta }$ is the initial value of the intracellular neutral solute molar density, $\beta \in S_{\beta }$, and

(21d) $${ℛ}_{\beta }(t):={𝒫}_{\beta }(t)-{𝒞}_{\beta }(t)\;\beta \in S_{\beta },t>0,$$

is the net production rate of the solute β (units: mMs^−1^).

#### 2.6.5. Time Evolution of Charged Solutes

The time evolution of the charged solute molar density is described by the mass balance equation in an integral form:

(22) $$\int_{\Omega_{t}}\frac{\partial c_{\alpha }}{\partial t}d\Omega_{t}=-\int_{\partial \Omega_{t}}j_{\alpha ,n}d\left(\partial \Omega_{t}\right)+\int_{\Omega_{t}}R_{\alpha }d\Omega_{t}\;\alpha \in S_{\alpha },$$

where $j_{\alpha ,n}$ is the normal molar flux density of $c_{\alpha }$ on the cell surface ${𝒮}_{t}$, and

(23) $$R_{\alpha }(x,t):=P_{\alpha }(x,t)-C_{\alpha }(x,t)\;x\in \Omega_{t},t\geq 0$$

is the net production rate of the intracellular ion α, with $P_{\alpha }$ and $C_{\alpha }$ being the production and consumption rates, respectively (units: mMs^−1^).

##### Assumption 10.

*We assume that*

(24a) $$c_{\alpha }(x,t)=c^{\alpha }(t)\Phi (x)\;x\in \Omega_{t},t\geq 0,$$

(24b) $$j_{\alpha ,n}(x,t)=j_{\alpha ,n}^{ch}(t)\eta_{\alpha }^{ch}(x)+j_{\alpha ,n}^{exch}(t)\eta_{\alpha }^{exch}(x)+j_{\alpha ,n}^{pump}(t)\eta_{\alpha }^{pump}(x)\;x\in \partial \Omega_{t},t\geq 0,$$

*where* $c^{\alpha }$ *is the time-dependent intracellular molar density of the ion* $\alpha \in S_{\alpha }$ *; the functions* $j_{\alpha ,n}^{ch}$ *and* $j_{\alpha ,n}^{exch\;}$ *are time-dependent normal molar flux densities on the cell surface representing passive ion transport and exchange, respectively; the function* $j_{\alpha ,n}^{pump}$ *is a time-dependent normal molar flux density on the cell surface representing ion exchange through active pumps; and the shape functions* $\;\eta_{\alpha }^{ch},\;\eta_{\alpha }^{exch}$, *and* $\eta_{\alpha }^{pump}$ *are such that*

(24c) $$\int_{\partial \Omega_{t}}\eta_{\alpha }^{ch}\left(x\right)\;d\left(\partial \Omega_{t}\right)=\Phi_{\alpha }^{ch}{𝒮}_{t},$$

(24d) $$\int_{\partial \Omega_{t}}\eta_{\alpha }^{exch}\left(x\right)\;d\left(\partial \Omega_{t}\right)=\Phi_{\alpha }^{exch}{𝒮}_{t},$$

(24e) $$\int_{\partial \Omega_{t}}\eta_{\alpha }^{pump}\left(x\right)\;d\left(\partial \Omega_{t}\right)=\Phi_{\alpha }^{pump}{𝒮}_{t},$$

*where* $\Phi_{\alpha }^{ch}$, $\Phi_{\alpha }^{exch}$, *and* $\Phi_{\alpha }^{pump}$ *are the surface fractions of the membrane protein associated with the passive transport, passive exchange, and active pump-mediated exchange of the ion* α, *respectively. We also assume that equations similar to (8) apply to* $P_{\alpha }$ *and* $C_{\alpha }$.

Using Assumption 10 in (22), we obtain the following reduced-order model for each charged solute molar density:

(25a) $$\frac{dc^{\alpha }(t)}{dt}=-\gamma j_{\alpha ,n}^{tot}(t)(𝒱(t){)}^{-1/3}+{ℛ}_{\alpha }(t)\;\alpha \in S_{\alpha },t>0,$$

(25b) $$j_{\alpha ,n}^{tot\;}(t):=\left[\Phi_{\alpha }^{ch\;}j_{\alpha ,n}^{ch\;}(t)+\Phi_{\alpha }^{exch\;}j_{\alpha ,n}^{exch\;}(t)+\Phi_{\alpha }^{pump\;}j_{\alpha ,n}^{pump\;}(t)\right]\;\alpha \in S_{\alpha },t>0,$$

(25c) $$c^{\alpha }(0)=c_{0}^{\alpha },$$

where $c_{0}^{\alpha }$ is the initial value of the intracellular charged solute molar density, $\alpha \in S_{\alpha }$, and

(25d) $${ℛ}_{\alpha }(t):={𝒫}_{\alpha }(t)-{𝒞}_{\alpha }(t)\;\alpha \in S_{\alpha },t>0,$$

is the net production rate of the ion α (units: mMs^−1^).

#### 2.6.6. Time Evolution of Membrane Potential

For any t≥0, the membrane potential is defined as

(26) $$\psi_{m}(t):=\psi^{in}(t)-\psi^{out}(t).$$

The time evolution of the membrane potential is described by the charge balance equation in an integral form:

(27) $$\int_{\partial \Omega_{t}}-\varepsilon_{m}\frac{\partial }{\partial t}\left(\frac{\partial \psi }{\partial n}\right)\;d\left(\partial \Omega_{t}\right)=-\int_{\partial \Omega_{t}}J_{cond,n}d\left(\partial \Omega_{t}\right)$$

where $\frac{\partial \psi }{\partial n}$ and $J_{cond,n}$ are the normal derivative of the electric potential and the normal total conduction current density on the cell surface ${𝒮}_{t}$, respectively.

##### Assumption 11.

**Figure 6.**
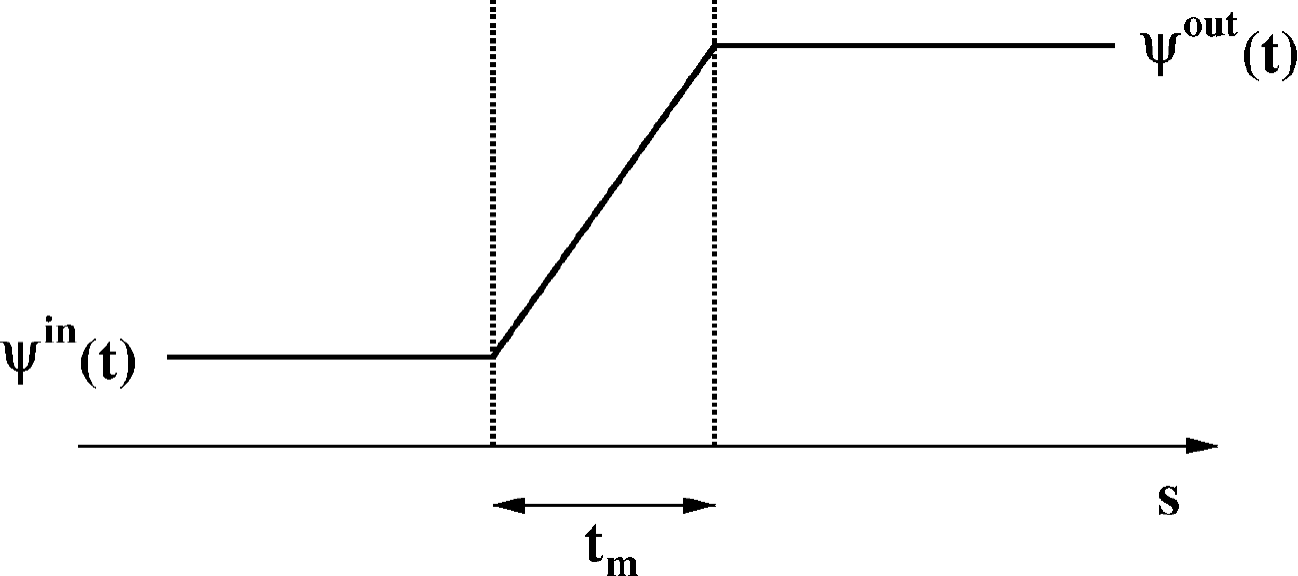
Transmembrane electric potential.

*We assume that the electric potential is a piecewise linear continuous function across the cell membrane thickness (see* Figure 6).

Using Assumption 11 in the left-hand side of (27), we obtain

(28) $$\int_{\partial \Omega_{t}}-\varepsilon_{m}\frac{\partial }{\partial t}\left(\frac{\partial \psi }{\partial n}\right)d\left(\partial \Omega_{t}\right)=c_{m}\frac{d\psi_{m}\left(t\right)}{dt}\Phi^{lip}𝒮\left(t\right)\;t\geq 0,$$

where

(29) $$c_{m}≔\frac{\varepsilon_{m}}{t_{m}}$$

is the cell specific capacitance (units: F m^−2^). The right-hand side of (27) is given by the following expression:

(30) $$-\int_{\partial \Omega_{t}}J_{cond,n}d\left(\partial \Omega_{t}\right)=-I_{cond}^{tot}\left(t\right)\;t\geq 0,$$

where $I_{cond\;}^{tot\;}$ is the total conduction current flowing across the cell membrane at the time t (units: A), defined as

(31) $$I_{cond\;}^{tot\;}(t)=\left[\sum_{\alpha \in S_{\alpha }}Fz_{\alpha }j_{\alpha ,n}^{tot\;}(t)\right]𝒮(t)\;t\geq 0,$$

Replacing (28) and (30) in (27), we obtain the following reduced-order model for the membrane potential:

(32a) $$\frac{d\psi_{m}(t)}{dt}=-{\left(\Phi^{lip}c_{m}\right)}^{-1}j_{n}^{\psi }(t)\;t>0,$$

(32b) $$j_{n}^{\psi }\left(t\right):=j_{cond,n}^{tot}\left(t\right)\;t>0,$$

(32c) $$\psi_{m}(0)=\psi_{m,0},$$

where

(32d) $$j_{cond,n}^{tot\;}(t)≔\sum_{\alpha \in S_{\alpha }}Fz_{\alpha }j_{\alpha ,n}^{tot\;}(t)\;t\geq 0$$

is the normal total conduction current density over the cell surface defined in (25b), and $\psi_{m,0}$ is the initial value of the membrane potential.

### 2.7. Compact Form of Reduced-Order Cell Model

Let us introduce the vector of dependent variables:

(33) $$Y(t):=\left[\begin{array}{c} 𝒱(t)\\ c_{\beta }(t)\\ c_{\alpha }(t)\\ \psi_{m}(t) \end{array}\right]\;t\geq 0,$$

where

(34) $$c_{\beta }(t):={\left[c_{\beta ,1}(t),\ldots ,c_{\beta ,N_{\beta }}(t)\right]}^{T}\;t\geq 0,$$

(35) $$c_{\alpha }(t):={\left[c_{\alpha ,1}(t),\ldots ,c_{\alpha ,N_{\alpha }}(t)\right]}^{T}\;t\geq 0,$$

are the column vectors containing the time values of the neutral and charged solute molar densities.

Let us define the vector of source terms:

(36) $$F(t,Y(t)):=\left[\begin{array}{l} -\gamma j_{v,n}(t)(𝒱(t){)}^{2/3}+(k_{w,prod}(t)-k_{w,cons}(t))𝒱(t)\\ -\gamma j_{\beta ,n}^{tot\;}(t)(𝒱(t){)}^{-1/3}+\left(P_{\beta }(t)-C_{\beta }(t)\right)\\ -\gamma j_{\alpha ,n}^{tot\;}(t)(𝒱(t){)}^{-1/3}+\left(P_{\alpha }(t)-C_{\alpha }(t)\right)\\ -{\left(c_{m}\Phi^{lip}\right)}^{-1}j_{n}^{\psi }(t) \end{array}\right]\;t\geq 0,$$

where $j_{\beta ,n}^{tot\;}$ and $j_{\alpha ,n}^{tot\;}$ are column vectors of the sizes $N_{\beta }$ and $N_{\alpha }$, respectively, containing the time values of the neutral and charged solute normal molar flux densities, whereas $P_{\beta }$ (and $C_{\beta }$ ) and $P_{\alpha }$ (and $C_{\alpha }$ ) are column vectors of the sizes $N_{\beta }$ and $N_{\alpha }$, respectively, containing the time values of the intracellular neutral and charged solute production (and consumption) terms.

Finally, let us define the column vector containing the initial condition of the model:

(37) $$Y_{0}:=\left[\begin{array}{c} {𝒱}_{0}\\ c_{0}^{\beta }\\ c_{0}^{\alpha }\\ \psi_{m,0} \end{array}\right],$$

where $c_{0}^{\beta }$ and $c_{0}^{\alpha }$ are column vectors of the sizes $N_{\beta }$ and $N_{\alpha }$, respectively, which contain the values of the initial conditions for the intracellular neutral and charged solute molar densities.

The equation system constituting the model of cell volume motion can be written in a compact form as

(38a) $$\frac{dY(t)}{dt}=F(t,Y(t))\;t>0,$$

(38b) $$Y\left(0\right)=Y_{0}.$$

System (38) comprises differential equations and algebraic equations, represented by the constitutive laws for the normal molar flux densities of neutral and charged solutes and for the production and consumption rates of water and solutes. The expressions of the normal fluid velocity on the cell surface and normal molar flux densities are provided in Appendix A whereas the expressions of the production and consumption rates are provided in Appendix B.

### 2.8. Numerical Approximation

The mathematical model proposed in this article was implemented within a computational virtual laboratory (CVL) for the simulation of cell volume motion.

The θ -method (see [19] (Chapeter 3)) and the Matlab tool ode suite were used for the numerical approximation of (38) (see [37]). In the case of the θ -method, the values θ=1 and θ=0.5 were utilized, with θ=1 corresponding to the Backward Euler (BE) method and θ=0.5 corresponding to the Crank–Nicolson (CN) method. In the case of the Matlab tool ode suite, the functions ode15s or ode23tb were utilized because they are specifically tailored to solve stiff problems like the object of the present article (see [38] (Chapter 11)). The function ode15s is endowed with a variable-order Backward Differentiation Formulae (BDF) method, whereas the function ode23tb uses the trapezoidal rule coupled with a BDF of the order 3.

The CVL allows the user to consider physical situations of increasing complexity, starting from the solution of the sole Cauchy problem (17) in which the fluid velocity is a given function of time and the production and consumption terms are functions of the time and cell volume. Simulation complexity can be increased by adding charged and neutral solutes, intracellular reactions, and transmembrane ion exchange mechanisms, as well as the presence of impermeant protein charges in both intra- and extracellular regions. This hierarchy of scenarios is investigated in Section 3 where the accuracy and reliability of the model predictions is verified against analytical solutions and available data.

## 3. Results

In this section, we validate the proposed CVL through the solution of case studies characterized by increasing complexity. In Section 3.1, we consider a reduced version of the model described in the previous pages, solely accounting for the cell volume equation. In Section 3.2, we use the full model and the concept of the “equivalent cell” introduced in Section 2.2 to simulate the process of AH production.

### 3.1. The Basic Configuration

This case study is mathematically represented by the sole model for cell volume motion (17).

#### Assumption 12.

*We make the following assumptions:*

1. $v_{p,n}(t)=\overset{‾}{v}$ *for all* t≥0, *with* $\overset{‾}{v}$ *being a given constant*;
2. $k_{w,prod}(t)=\kappa_{a}$ *for* t≥0, *with* $\kappa_{a}$ *being a given positive constant (units*: s^−1^);
3. $k_{w,\;cons}(t)=\kappa_{d}\frac{𝒱(t)}{{𝒱}_{ref\;}}$ *for* t≥0, *with* $\kappa_{d}$ (*units*: s^−1^) *and* ${𝒱}_{ref}$ (*units*: m^3^*) being given positive constants.*

Replacing the assumptions about the water volume production and consumption rates in (17d), we obtain

(39) $${ℛ}_{w}(t)=\kappa_{a}-\kappa_{d}\frac{𝒱(t)}{{𝒱}_{ref}}\;t\geq 0,$$

Replacing Assumption 12 and (39) in (17a), we obtain the following Cauchy problem:

(40a) $$\frac{d𝒱(t)}{dt}=-\gamma \Phi^{AQP}\overset{‾}{v}(𝒱(t){)}^{2/3}+\left(\kappa_{a}-\kappa_{d}\frac{𝒱(t)}{{𝒱}_{ref\;}}\right)𝒱(t)\;t>0,$$

(40b) $$𝒱(0)={𝒱}_{ref},$$

where ${𝒱}_{ref\;}=(4\pi /3)R_{cell}^{3}$ is the cell volume in resting conditions.

The equilibrium points of System (40) are the solution of the following nonlinear algebraic equation:

(41) $$f(x)=-\gamma \Phi^{AQP}\overset{‾}{v}x^{2/3}+{𝒱}_{ref}^{1/3}x\left(\kappa_{a}-\kappa_{d}x\right)=0,$$

where $x:={𝒱}_{\infty }/{𝒱}_{\mathrm{ref}}$ is the dependent variable and ${𝒱}_{\infty }$ is the stationary value of the cell volume.

**Figure 7.**
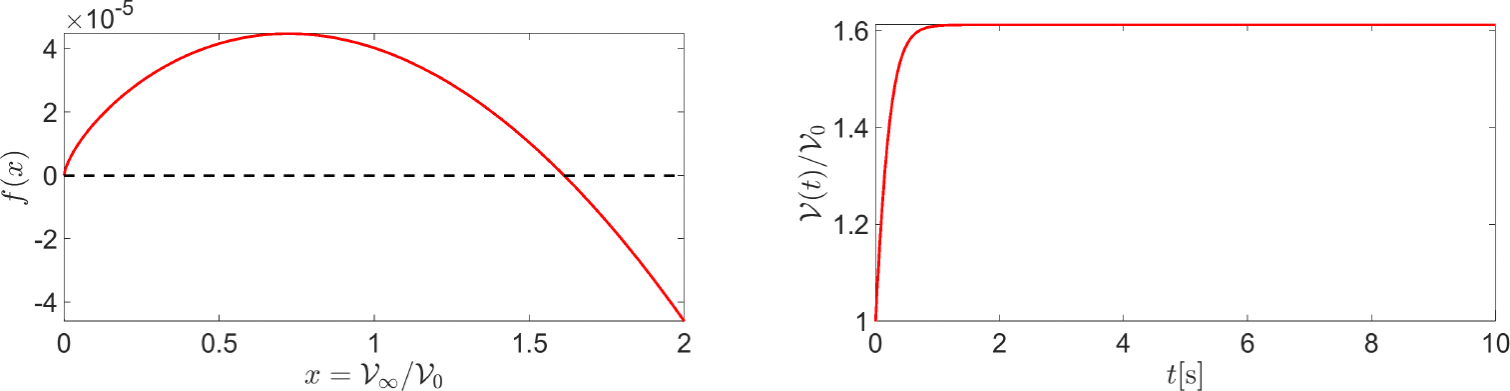
**Left** panel: a plot of f(x). **Right** panel: a plot of $𝒱(t)/{𝒱}_{\mathrm{ref}}$ in the time interval [0, 10] s. The values of the input data are as follows: $R_{\mathrm{cell}}=10\times 10^{-6}\;m$, $\Phi^{AQP}=0.5$ ; $\overset{‾}{v}=-30\times 10^{-6}\;ms^{-1}$ ; $\kappa_{a}=1{\;s}^{-1}$ ; and $k_{d}=3{\;s}^{-1}$.

Figure 7 (left panel) shows a graph of f, corresponding to the following values of the input data: $R_{\mathrm{cell}}=10\cdot 10^{-6}\;m$ ; $\Phi^{AQP}=0.5$ ; $\overset{‾}{v}=-30\cdot 10^{-6}\;ms^{-1}$, $\kappa_{a}=1{\;s}^{-1}$ ; and $k_{d}=3{\;s}^{-1}$. System (40) admits two equilibrium points: ${𝒱}_{\infty ,1}=0{\;m}^{3}$ and ${𝒱}_{\infty ,2}=1.6124{𝒱}_{\mathrm{ref}}=6.754\cdot 10^{-15}{\;m}^{3}$. We have

$${\left.\frac{df}{dx}\right|}_{x=0}=+\infty ,{\left.\;\frac{df}{dx}\right|}_{x=1.6124}=-9.86\cdot 10^{-5}\;ms^{-1},$$

from which we can conclude that ${𝒱}_{\infty ,2}$ is the only stable equilibrium point of System (40). The theoretical conclusions of the stability analysis are confirmed by Figure 7 (left panel) which shows the normalized cell volume computed by solving System (40) in the time interval [0, 10] s with the following values of the model parameters: $R_{\mathrm{cell}}=10\cdot 10^{-6}\;m$ ; $\Phi^{AQP}=0.5$ ; $\overset{‾}{v}=-30\cdot 10^{-6}\;ms^{-1}$ ; $\kappa_{a}=1{\;s}^{-1}$ ; and $k_{d}=3{\;s}^{-1}$. Cell volume dynamics were studied using the BE method with a uniform time partition made of 10^5^ elements. Consistent with the physical configuration illustrated in Figure 3, the cell tends to increase its volume by more than 60% because of the inflow of water from the extracellular region. The cell volume reaches a stationary limit because of intracellular water consumption dominating over intracellular water production.

**Figure 8.**
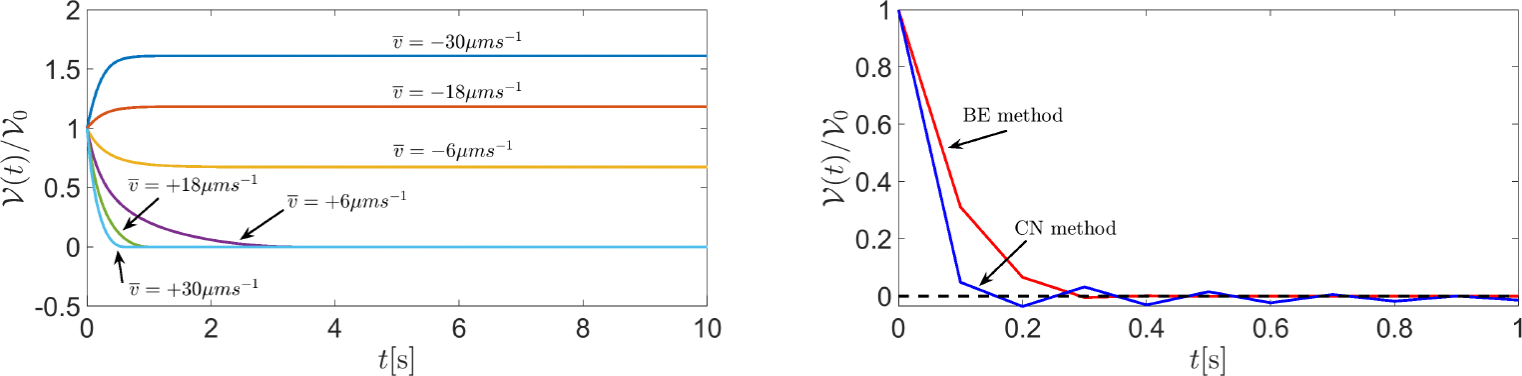
**Left** panel: a plot of $𝒱(t)/{𝒱}_{\mathrm{ref}}$ in the time interval [0, 1] s. The value of fluid velocity (expressed in μms^−1^) is indicated for each computed normalized cell volume. **Right** panel: a plot of $𝒱(t)/{𝒱}_{\mathrm{ref}}$ in the time interval [0, 1] s.

Figure 8 (left panel) illustrates the temporal evolution of cell volume in the case where the values of $\overset{‾}{v}$ are [−30 − 18, −6, +6, +18, +30] × 10^−6^ ms^−1^. The results suggest that the cell switches between swelling and shrinking as fluid velocity changes its sign from negative to positive. Interestingly, the BE method gives rise to positive cell volumes for each t≥0 and for each considered value of $\overset{‾}{v}$. This outcome is the consequence of the positivity principle enjoyed by the BE method when applied to the linear equation $y^{\prime }(t)=-\lambda y(t)$ for t≥0, with λ being a given positive constant. The difference between using the BE method (θ=1) and another θ -method with θ∈[0,1) is illustrated in Figure 8 (right panel) which shows a comparison between the BE method (red line) and the Crank-Nicolson method (CN, blue line) in the solution of (40) when the time interval is [0, 1] s, the fluid velocity is $\overset{‾}{v}=100\times 10^{-6}\;ms^{-1}$, and the number of time elements is 10, corresponding to the time step Δt=0.1s. The values of the remaining model parameters are the same as in the previous example. The results indicate that the solution computed by the CN method is affected by spurious unphysical oscillations, unlike that computed by the BE method. Such oscillations can be removed by increasing the number of discretization time elements, at the price of increasing the computational effort.

**Figure 9.**
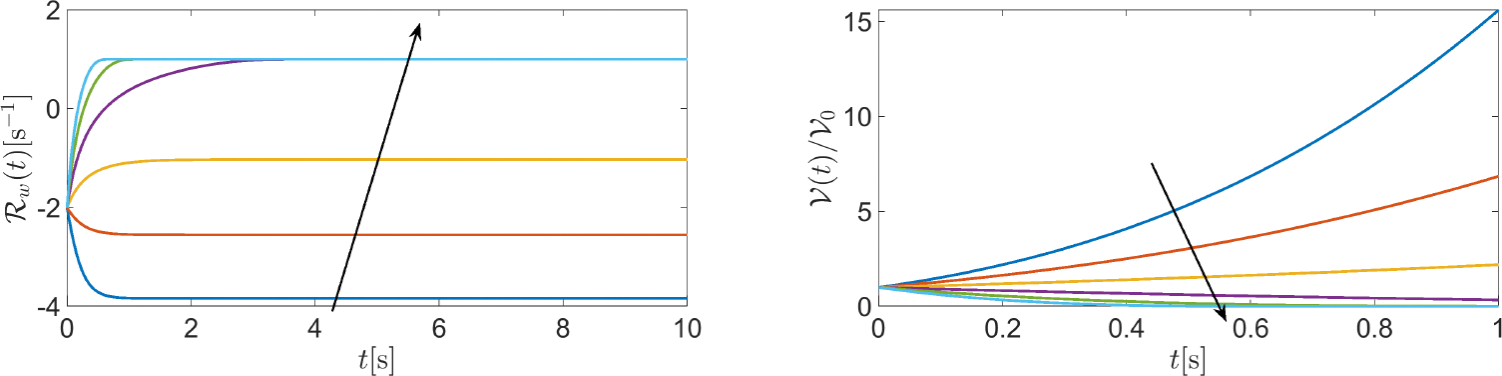
**Left** panel: a plot of the water volume net production rate ${ℛ}_{w}(t)$ in the time interval [0, 10] s. **Right** panel: a plot of $𝒱(t)/{𝒱}_{\mathrm{ref}}$ in the time interval [0, 1] s in the case where ℛ(t)=0 for every $t\in \left[0,\;1\right]\;s$. Fluid velocity varies in the range [−30 + 30] × 10^−6^ ms^−1^. The arrow indicates velocity increase from negative to positive values.

Figure 9 (left panel) illustrates the temporal evolution of the water volume net production rate ${ℛ}_{w}$ in the time interval [0, 10] s with the fluid velocity varying in the range [−30 + 30] · 10^−6^ ms^−1^. We see that the stationary value of ${ℛ}_{w}$, for each considered normal fluid velocity in the range [−30 + 30] · 10^−6^ ms^−1^, changes from negative to positive, with the magnitude for $v_{n}=-30\cdot 10^{-6}\;ms^{-1}$ being three times larger than for $v_{n}=+30\cdot 10^{-6}\;ms^{-1}$. Figure 9 (right panel) illustrates the temporal evolution of the cell normalized volume in the time interval [0, 1] s (ten times smaller than before) in the case where ℛ(t)=0 for t≥0 and the fluid velocity varies in the range [−30 + 30] · 10^−6^ ms^−1^. We see that in the absence of intracellular regulatory mechanisms, the model System (40) predicts an abnormal increase in cell volume for a highly negative value of normal fluid velocity.

### 3.2. Cell Homeostasis in the Ciliary Epithelium of the Eye

This case study is mathematically represented by the Cauchy problem (38) in which the cell represents one of the pigmented/nonpigmented couplets in the ciliary epithelium (CE) of the ciliary body of the eye (see [7,8,39]). The aim of this study is to apply the model proposed in this article to characterize the homeostatic configuration of the cell under the physiological conditions of the system. According to the data reported in [7], such conditions correspond to the following:

**C1.** The volume of the CE, equal to 8 μL;

**C2.** The number of cell couplets, $N_{\mathrm{cells,CE}}$, constituting the CE, equal to 4 million;

**C3.** The intraocular pressure, equal to 15 mmHg;

**C4.** The AH volumetric flow rate, equal to 2.75 μL min^−1^.

#### Assumption 13.

*We make the following assumptions:*

1. *The considered sets of the neutral and charged solutes are*
(42) $$S_{\beta }=\left\{{CO}_{2},H_{2}{CO}_{3}\right\},$$
(43) $$S_{\alpha }=\left\{{Na}^{+},K^{+},H^{+},{Cl}^{-},{HCO}_{3}^{-}\right\};$$
2. *The molar densities of the neutral and charged solutes are given constants denoted by* ${\overset{‾}{c}}_{\beta ,ex}$, $\beta \in S_{\beta }$, *and* ${\overset{‾}{c}}_{\alpha ,ex}$, $\alpha \in S_{\alpha }$ ;
3. *The hydraulic pressure difference is a given constant denoted by* $\Delta \overset{‾}{p}={\overset{‾}{p}}_{\mathrm{in}}-{\overset{‾}{p}}_{\mathrm{ex}}$, *where* ${\overset{‾}{p}}_{\mathrm{in}}$ *and* ${\overset{‾}{p}}_{ex}$ *are the values of the intracellular and extracellular fluid pressure, respectively;*
4. *No transmembrane ion exchangers are considered so that* $\Phi^{\mathrm{exch}}=0$ ;
5. *The model of carrier membrane proteins is described in* Appendix A.3;
6. *The model of ion channels is described in* Appendix A.4;
7. *The model of the net production rates for the neutral and charged solutes is described in* Appendix B.1;
8. *The model of the net production rate in cell volume regulation is described in* Appendix B.2.

The values of all model parameters that were used in the computer simulations illustrated in this section are listed in Table 1.

The vector containing the initial condition data is

$$Y_{0}={\left[2\times 10^{-15},\;0,\;0,\;10,\;140,\;5.0119\times 10^{-5},\;7,\;10^{-4},\;-8.0097\times 10^{-2}\right]}^{T}\;{units:\;\left[m^{3},mM,mM,mM,mM,mM,mM,mM,V\right]}^{T}.$$

The vectors containing the extracellular values of the solute molar density are

$${\bar{c}}_{\beta }={\left[0.5,\;1.3\cdot 10^{-3}\right]}^{T}\;uniys:\;[mM,mM{]}^{T},$$

$${\bar{c}}_{\alpha }={\left[130,\;5,\;3.1623\cdot 10^{-5},150,\;9.9\right]}^{T}\;units:\;[mM,mM,mM,mM,mM{]}^{T}.$$

The values of the remaining model parameters can be found in Appendices B.1, B.2 and B.3. Computations were performed using the Matlab solver ode15s, setting both relative and absolute tolerances equal to 10^−12^.

#### 3.2.1. Electroneutrality and Impermeant Charged Proteins

Electroneutrality is essential in the maintainance of cell volume as a function of time and of phenomena occurring in the intracellular and extracellular regions (see [3] and references cited therein). The computation of the total electric charge densities $\rho_{\mathrm{mob,in}}$ and $\rho_{\mathrm{mob,ex}}$ due to mobile ions inside and outside the cell in resting conditions yields the following:

$$\rho_{\mathrm{mob,in}}=F\sum_{\alpha \in S_{\alpha }}z_{\alpha }c_{\alpha ,\mathrm{in}}\left(0\right)=1.3797\times 10^{7}{Cm}^{-3},$$

$$\rho_{\mathrm{mob,ex}}=F\sum_{\alpha \in S_{\alpha }}z_{\alpha }{\overset{‾}{c}}_{\alpha }=-9.5519\times 10^{5}\;{Cm}^{-3}.$$

These results indicate that the intracellular region (at t=0 ) is characterized by a high excess of positive charge whereas the extracellular region (at t=0 ) is characterized by a high excess of negative charge. This charge difference gives rise to a large osmotic pressure difference across the cell membrane which may eventually lead to the disruption of cell integrity. To neutralize the excess of positive and negative charge, we need the presence of internally sequestered impermeant charges inside and outside the cell. Denoting by $c_{X}$ and $z_{X}$ the molar density and charge number of the impermeant charge, we have

(44a) $$c_{X,\mathrm{in}}=143\;mM\;z_{X,\mathrm{in}}=-1,$$

(44b) $$c_{X,ex}=9.9\;mM\;z_{X,ex}=+1.$$

These results indicate that a high molar density of fixed anions is sequestered inside the cell cytoplasm whereas a much smaller molar density of cations is required to make the extracellular solution electroneutral.

##### Assumption 14.

*We assume that the number of moles of the intracellular impermeant charge* $n_{X}$ *is constant during the time evolution of the cell*.

Let $c_{X}(0)$ denote the intracellular molar density of the impermeant charge at the time t=0 (units: mM). By the definition of molar density, we have

(45) $$c_{X}(0)=\frac{n_{X}}{{𝒱}_{0}}.$$

Using Assumption 14 and (45), we can express the intracellular impermeant charge molar density for any time, t≥0, as

(46) $$c_{X,\mathrm{in}\;}(t)=\frac{n_{X}}{𝒱(t)}=c_{X}(0)\frac{{𝒱}_{0}}{𝒱(t)}\;t\geq 0.$$

The simulations illustrated in the next sections were conducted using the values of $z_{X}$ and $c_{X}$ in (44) and the constitutive Equation (46).

#### 3.2.2. Fast-Time-Scale Cell Evolution

We investigated the evolution of the cell in the time interval $\left[t_{0},t_{\mathrm{end}}\right]$, where $t_{0}=0\;s$ and $t_{\mathrm{end}}=50\times 10^{-12}\;s$.

**Figure 10.**
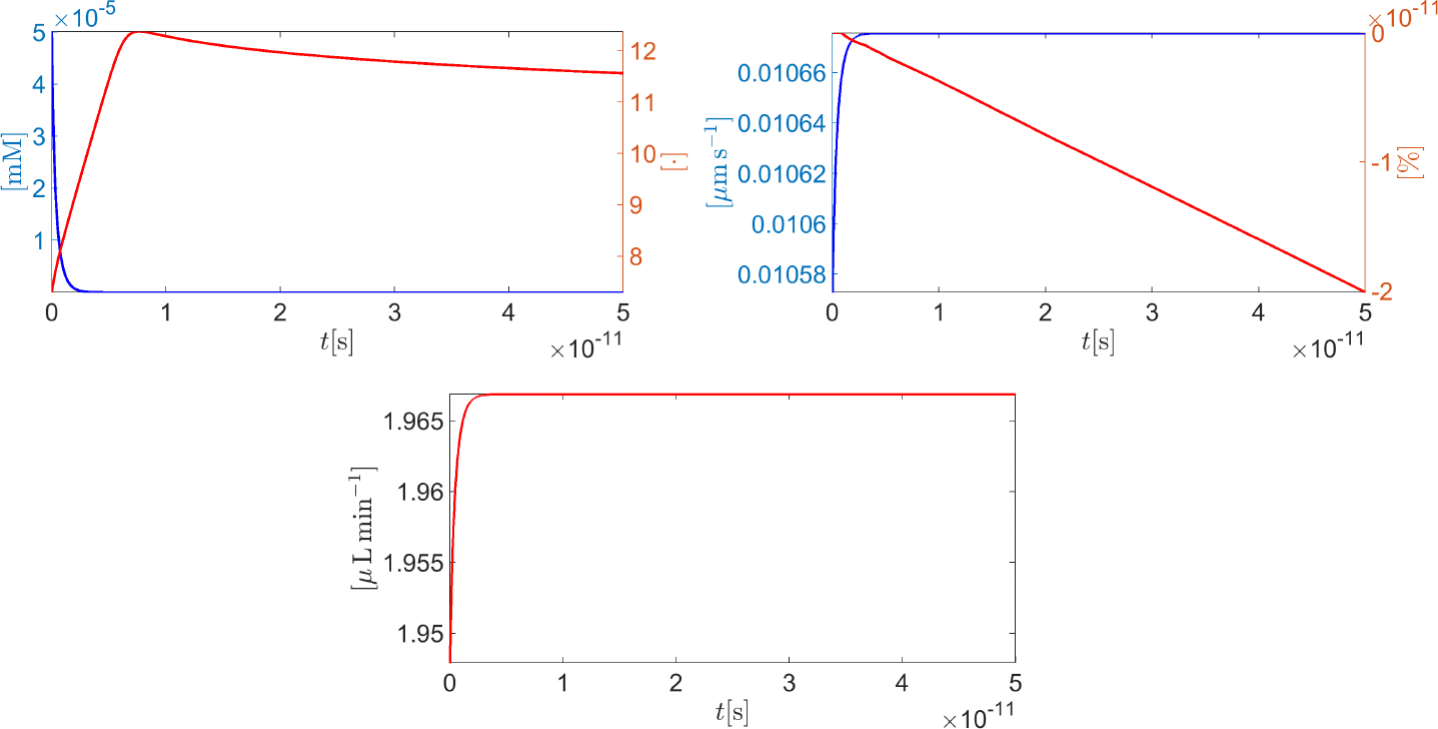
Left panel: blue curve, $c_{H^{+},in}(t)$ ; red curve, $pH_{,\mathrm{in}}\left(t\right),t\in \left[0,\;50\cdot 10^{-12}\right]\;s$. **Right** panel: blue curve, $v_{\mathrm{cell},n}(t)$ ; red curve, $\Delta {𝒱}_{\%}\left(t\right)$, $t\in \left[0,\;50\cdot 10^{-12}\right]\;s$. Middle panel (bottom): total AH volumetric flow rate $Q_{AH}(t)$ for $t\in \left[0,\;50\cdot 10^{-12}\right]\;s$.

Figure 10 (left panel) illustrates the time evolution of the intracellular protonated hydrogen (blue curve) and the corresponding intracellular pH (red curve). Hydrogen fast diffusion from the intracellular side into the extracellular side determines a sharp decrease in $c_{H^{+},\mathrm{in}}$ so that the cytoplasm solution turns into a very basic condition (the maximum value of the intracellular pH is 12.5). Figure 10 (right panel) illustrates the time evolution of the average cell normal surface velocity (blue curve) and the corresponding percentage variation, $\Delta {𝒱}_{\%}$, in the cell volume with respect to the initial condition (red curve). The cell surface velocity is positive and very small in magnitude (less than 0.02 μm s^−1^) so the volume of the cell experiences a very little decrease (maximum magnitude equal to 2 × 10^−11^%) with respect to resting conditions. Figure 10 (middle panel, bottom) illustrates the time evolution of the total AH volumetric flow rate $Q_{AH}$ throughout the CE (units: μL min^−1^). The quantity $Q_{AH}(t)$ is computed for every $t\in \left[t_{0},t_{\mathrm{end}}\right]$ using the following relation

(47) $$Q_{AH}\left(t\right)=\left(N_{\mathrm{cells},CE}v_{\mathrm{cell}\text{,}n}\left(t\right)𝒮\left(t\right)\right)60\times 10^{9}\;units:\;\mu L{\;min}^{-1}\;t\in \left[t_{0},t_{end}\right].$$

In less than 50 ps, $Q_{AH}$ reaches almost 72% of the volumetric flow rate that is physiologically expected in a normal-tension individual (see condition C4).

#### 3.2.3. Medium-Time-Scale Cell Evolution

We investigated the evolution of the cell in the time interval $\left[t_{0},t_{\mathrm{end}}\right]$, where $t_{0}=0\;s$ and $t_{\mathrm{end}}=5\;s$.

**Figure 11.**
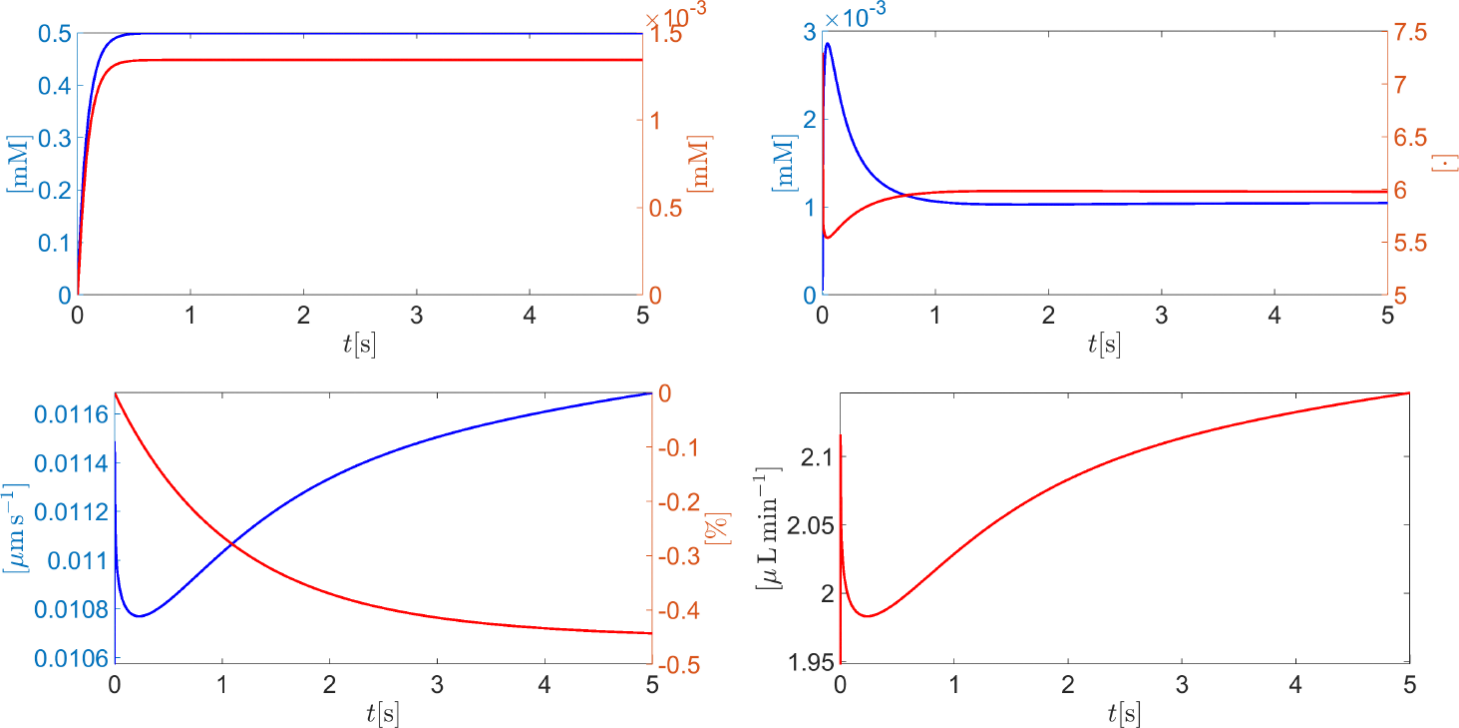
**Top left** panel: blue curve, $c_{{CO}_{2,in}}(t)$ ; red curve, $H_{2}{CO}_{3,in}\left(t\right)$, $t\in \left[0,\;5\right]\;s$. **Top right** panel: blue curve, $H_{\mathrm{in}}^{+}(t)$ ; red curve, ${pH}_{\mathrm{in}}(t)$, $t\in \left[0,\;5\right]\;s$. **Bottom left** panel: blue curve, average cell normal surface velocity, $v_{\mathrm{cell}\text{,}n}(t)$ ; red curve, percentage volume variation, $\Delta {𝒱}_{\%}\left(t\right)$, $t\in \left[0,\;5\right]\;s$. **Bottom right** panel: total AH volumetric flow rate $Q_{AH}(t)$ for $t\in \left[0,\;5\right]\;s$.

Figure 11 (top left panel) illustrates the time evolution of the intracellular CO_2_ (blue curve) and H_2_CO_3_ (red curve) molar densities. In less than 0.5 s, the hydration process gives rise to a significant production of carbon dioxide and carbonic acid, which is eventually followed by a stationary condition corresponding to the dynamic equilibrium of the reaction (A8a). The consequence of the CO_2_ hydration can also be seen from Figure 11 (top right panel) which illustrates the time evolution of the intracellular $H^{+}$ molar density and the corresponding pH (blue and red curves, respectively). Protonated hydrogen concentration increases significantly until almost t=0.125s because of carbonic acid dissociation (forward reaction in (A8b)). Then, the association reaction (backward reaction in (A8b)) with bicarbonate gives rise to a decrease in $c_{H^{+},\mathrm{in}}$ until a stationary condition is reached. In such a condition, the value of the intracellular pH (almost 6) indicates that the cytoplasm solution is acidic. Figure 11 (bottom left panel) illustrates the time evolution of the average cell normal surface velocity (blue curve) and the corresponding percentage variation $\Delta V_{\%}$ of the cell volume with respect to the initial condition (red curve). As in the fast-scale cell evolution, the cell surface velocity is positive and very small in magnitude (less than 0.02 μm s^−1^). In this case, however, the much larger time duration of the analysis (5 s instead of 50 ps) allows the cell to decrease by a larger percentage amount with respect to resting conditions (less than 0.5% instead of 2 × 10^−11^%). Figure 11 (bottom right panel) illustrates the time evolution of the total AH volumetric flow rate $Q_{AH}$ throughout the CE (units: μL min^−1^). In 5 s, $Q_{AH}$ reaches more than 78% of the volumetric flow rate that is physiologically expected in a normal-tension individual.

#### 3.2.4. Long-Time-Scale Cell Evolution

We investigated the evolution of the cell in the time interval $\left[t_{0},t_{\mathrm{end}}\right]$, where $t_{0}=0\;s$ and $t_{\mathrm{end}}=5400\;s$. The value of $t_{\mathrm{end}}$ corresponds to the time that is needed by the eye to completely replace the AH content of the anterior chamber (see [7,9]).

**Figure 12.**
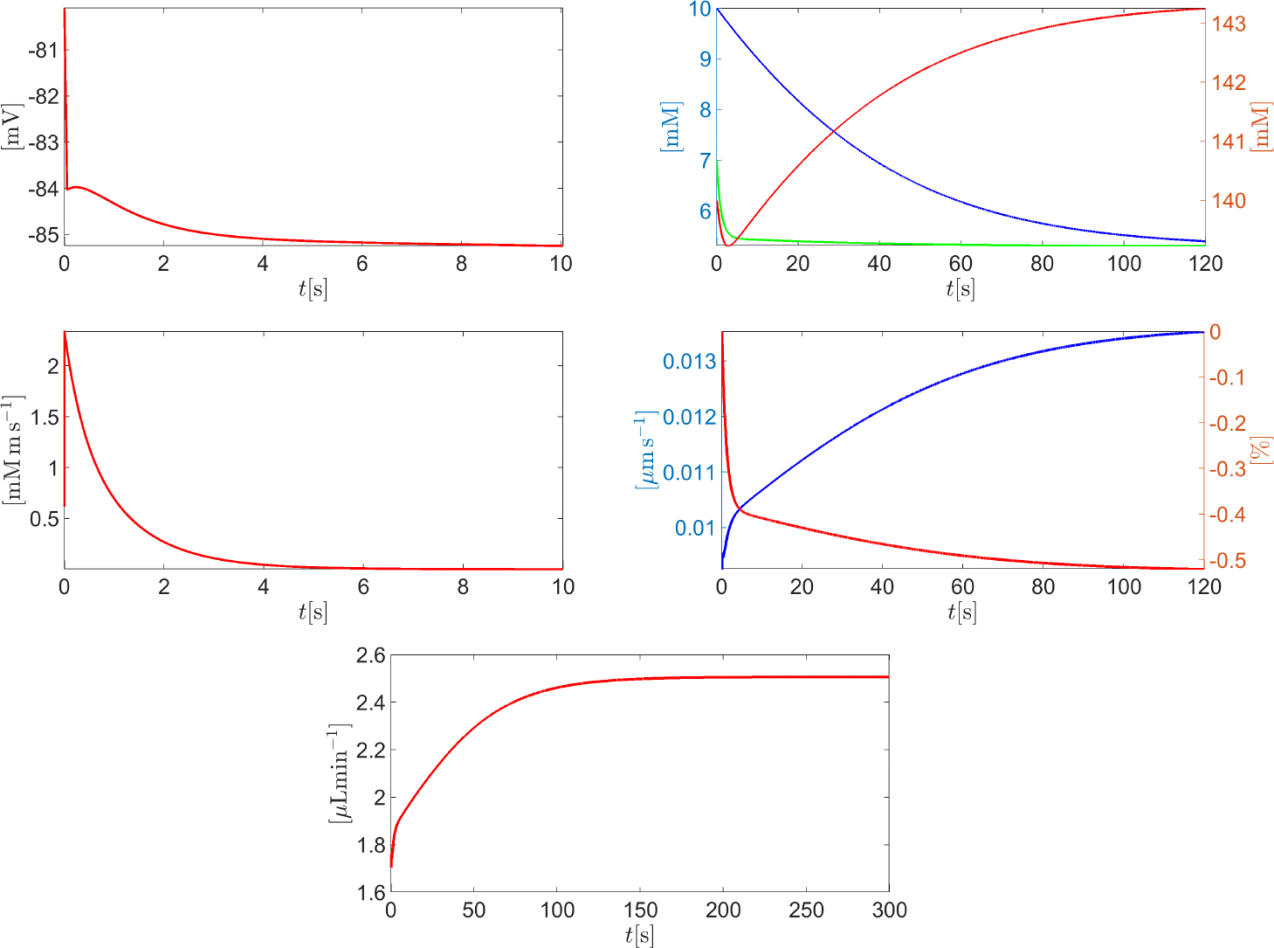
**Top left** panel: a zoom of the membrane potential $\psi_{m}(t)$ (units: mV) in the time interval $t\in \left[0,\;10\right]\;s$. **Top right** panel: a zoom of $c_{{Na}^{+},\mathrm{in}}(t)$ (blue curve), $c_{K^{+},\mathrm{in}}(t)$ (red curve), and $c_{{Cl}^{-},\mathrm{in}}(t)$ (green curve) (units: mM) in the time interval $t\in \left[0,\;120\right]\;s$. **Middle left** panel: a zoom of the chlorine molar flux density $j_{{Cl}^{-}}^{c*}(t)$ (units: mM ms^−1^) in the time interval $t\in \left[0,\;10\right]\;s$. **Middle right** panel: a zoom of $v_{\mathrm{cell},n}(t)$ (blue curve) and $\Delta {𝒱}_{\%}(t)$ (red curve) in the time interval $t\in \left[0,\;120\right]\;s$. **Bottom center** panel: a zoom of the total AH volumetric flow rate in the time interval $t\in \left[0,\;300\right]\;s$.

Figure 12 (top left panel) illustrates a zoomed view of the time evolution of the membrane potential in the time interval $t\in \left[0,\;10\right]\;s$. After an initial ultra-fast transient due to the mismatch between the intracellularly applied initial conditions and the conditions in the extracellular bath, the cell reaches a stationary state of −85.9 mV, corresponding to an after-hyperpolarization of −5.9 mV. Figure 12 (top right panel) illustrates a zoomed view of the time evolution of the intracellular molar densities of sodium (blue curve), potassium (red curve), and chlorine (green curve) for $t\in \left[0,\;120\right]\;s$. The concentration of sodium experiences a significant depletion (from 10 to 5.4 mM) because of the NaK ATPase pump activity. Similarly, the concentration of potassium increases from 140 mM up to a stationary value of almost 144 mM. The green curve in Figure 12 (top right panel) indicates that the concentration of intracellular chlorine experiences a depletion from 7 mM to 5.3 mM. This can be explained by Figure 12 (middle left panel) which illustrates a zoom of the chlorine molar flux density $j_{{Cl}^{-},n}(t)$ (units: mM ms^−1^) for $t\in \left[0,\;10\right]\;s$. The positive value of $j_{{Cl}^{-},n}$ indicates that chlorine is swept out of the cell cytoplasm with a progressively reducing magnitude over time. Figure 12 (middle right panel) illustrates a zoom of the time evolution of the average cell normal surface velocity (blue curve) and the corresponding percentage variation $\Delta {𝒱}_{\%}$ of the cell volume with respect to the initial condition (red curve) for $t\in \left[0,\;120\right]\;s$. As in the previous conditions, the cell surface velocity is positive and very small in magnitude (less than 0.015 μm s^−1^). The stationary cell volume percentage decrease with respect to resting conditions is less than 0.57%. Figure 12 (bottom center panel) illustrates a zoom of the time evolution of the total AH volumetric flow rate $Q_{AH}$ throughout the CE (units: μL min^−1^) for $t\in \left[0,\;300\right]\;s$. The stationary value of $Q_{AH}$ is 2.75017 μL min^−1^, with a percentage error of −0.0062% with respect to the physiological value of 2.75 μL min^−1^.

## 4. Discussion

The development of a mathematical model and of a CVL for the simulation of the process of the production, flow, and outflow of AH has been subject of investigation in recent years (see [15–18]). Our proposed formulation is characterized by the following features: (F.1) it is based on the use of homogeneous mixtures including neutral and charged solutes (see [19] (Chapter 13)); (F.2) it is defined at the level of the single cell; and (F.3) it utilizes a model reduction procedure from three spatial dimensions to zero spatial dimensions. Feature (F.1) confers a solid theoretical foundation to the proposed model. Features (F.2) and (F.3) make the model structure simple and the computational schemes fast and suitable for adoption in a clinical environment. The model is a consistent generalization of previous approaches [3,4] as it shares with them the same conceptual, simplifying assumption of working at the level of the single cell. This assumption is applied here to evaluate the collective behaviour of the cells in the CE of the eye in the process of the production of AH. The model that we propose in these pages also has limitations: (L.1) the dependence of all the variables from the spatial coordinate is neglected; (L.2) several important transmembrane mechanisms regulating solute exchange are not considered in the simulations; (L.3) the statistical variability of the parameters is not accounted for. Limitation (L.1) is a consequence of the use of the 3D-to-0D reduction procedure. Limitation (L.2) is a choice to prevent the proliferation of model parameters, thereby rendering the analysis of simulation predictions more easily. Limitation (L.3) is a consequence of the choice of using a mechanistic (continuum-based) approach. We intend to remove all these limitations in future extensions of the formulation considered in the present article.

In the next sections, we use the CVL developed in the present article to address specific questions of clinical importance in the study of AH production and its relation to ocular diseases. In Section 4.1, we assess the impact of oncotic pressure due to impermeant charge. In Section 4.2, we assess the impact of Na^+^/K^+^ ATPase. In Section 4.3, we assess the impact of the carbonic anhydrase enzyme. In Section 4.4, we assess the impact of IOP.

### 4.1. The Impact of Oncotic Pressure Due to Impermeant Charge

In this simulation, we solve System (38) in the time interval [0, 600] s with the same set of parameters as in Section 3.2, except the reflection coefficient $\sigma_{X}$ which is set equal to [0:0.2:1] (Matlab vector notation). By doing so, the weight of the contribution of the oncotic pressure difference (A5h) to the total osmo-oncotic pressure difference (A5k) increases progressively from $0\%\;\left(\sigma_{X}=0\right)$ to $100\%\;\left(\sigma_{X}=1\right)$, thereby allowing us to investigate the impact of oncotic pressure difference on the AH simulation.

**Figure 13.**
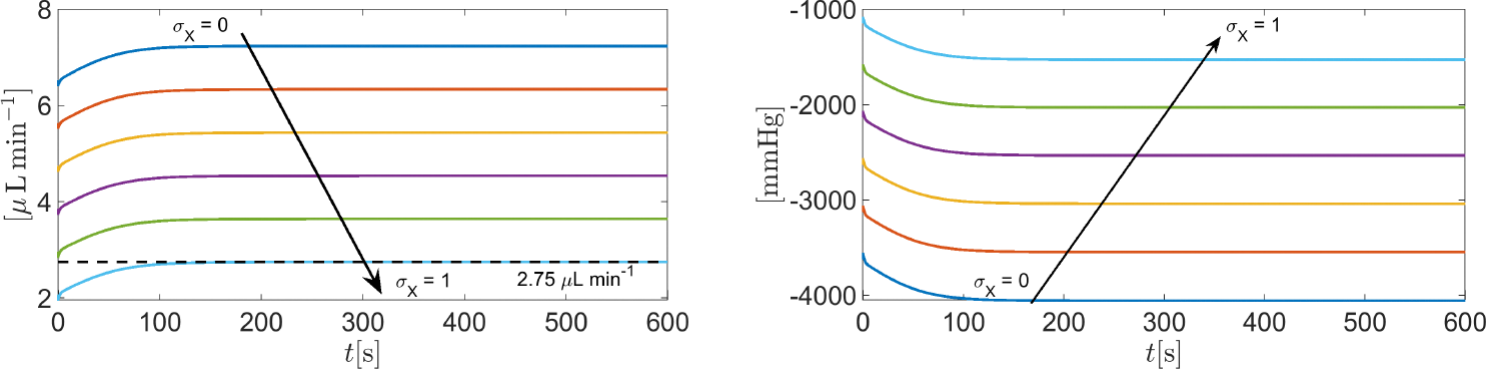
**Left** panel: total AH volumetric flow rate $Q_{AH}$ for $t\in \left[0,\;600\right]\;s$ as a function of $\sigma_{X}$. The black dashed line indicates the physiological value of $Q_{AH}$, equal to 2.75 μL min^−1^, when IOP=15mmHg. **Right** panel: the total osmo-oncotic pressure difference ΔΠ(t) for $t\in \left[0,\;600\right]\;s$ as a function of $\sigma_{X}$.

Figure 13 (left panel) illustrates the time evolution of the total volumetric flow rate $Q_{AH}(t)$ as a function of the time t in the interval $t\in \left[0,\;600\right]\;s$. The results indicate that the smaller $\sigma_{X}$ is, the larger the predicted total volumetric flow rate of AH is. In particular, the value of $Q_{\text{AH}}$ predicted by the model which is compatible with the given intracellular and extracellular fluid pressures ( ${\overset{‾}{p}}_{\mathrm{in}}=20\;mmHg$ and ${\overset{‾}{p}}_{\mathrm{ex}}=15\;mmHg$, respectively) is obtained for $\sigma_{X}=1$. To better understand the effect of properly including impermeant charge in AH modeling, we illustrate in Figure 13 (right panel) the time evolution of the total osmo-oncotic pressure difference ΔΠ(t) in the interval $t\in \left[0,\;600\right]\;s$. We see that the magnitude of ΔΠ largely exceeds the contribution from hydrostatic pressure difference $\Delta \overset{‾}{p}=5\;mmHg$ for every value of $\sigma_{X}\in [0:0.2:1]$. Moreover, for every $\sigma_{X}\in [0:0.2:1]$, the oncotic pressure difference $\Delta \Pi_{X}$ is always positive whereas the total osmotic pressure difference $\Delta \Pi_{osm}$ is always negative, so, as $\sigma_{X}$ increases, the magnitude of the total osmo-oncotic pressure difference decreases to reach the value of −1500 mmHg.

### 4.2. The Impact of Na^+^/K^+^ ATPase

In this simulation, we solve System (38) in the time interval [0, 5400] s with the same set of parameters as in Section 3.2, except the amplification coefficient $M_{ATP}$ in (A12e) which is set equal to [0:0.5:2] (Matlab vector notation). By doing so, the weight of the contribution of the sodium–potassium pump to the electrochemical balance of the cell increases progressively from $0\%\;\left(M_{ATP}=0\right)$ to $100\%\;\left(M_{ATP}=2\right)$, with respect to the working conditions of Section 3.2, thereby allowing us to investigate the impact of ${Na}^{+}/K^{+}$ ATPase on the AH simulation.

**Figure 14.**
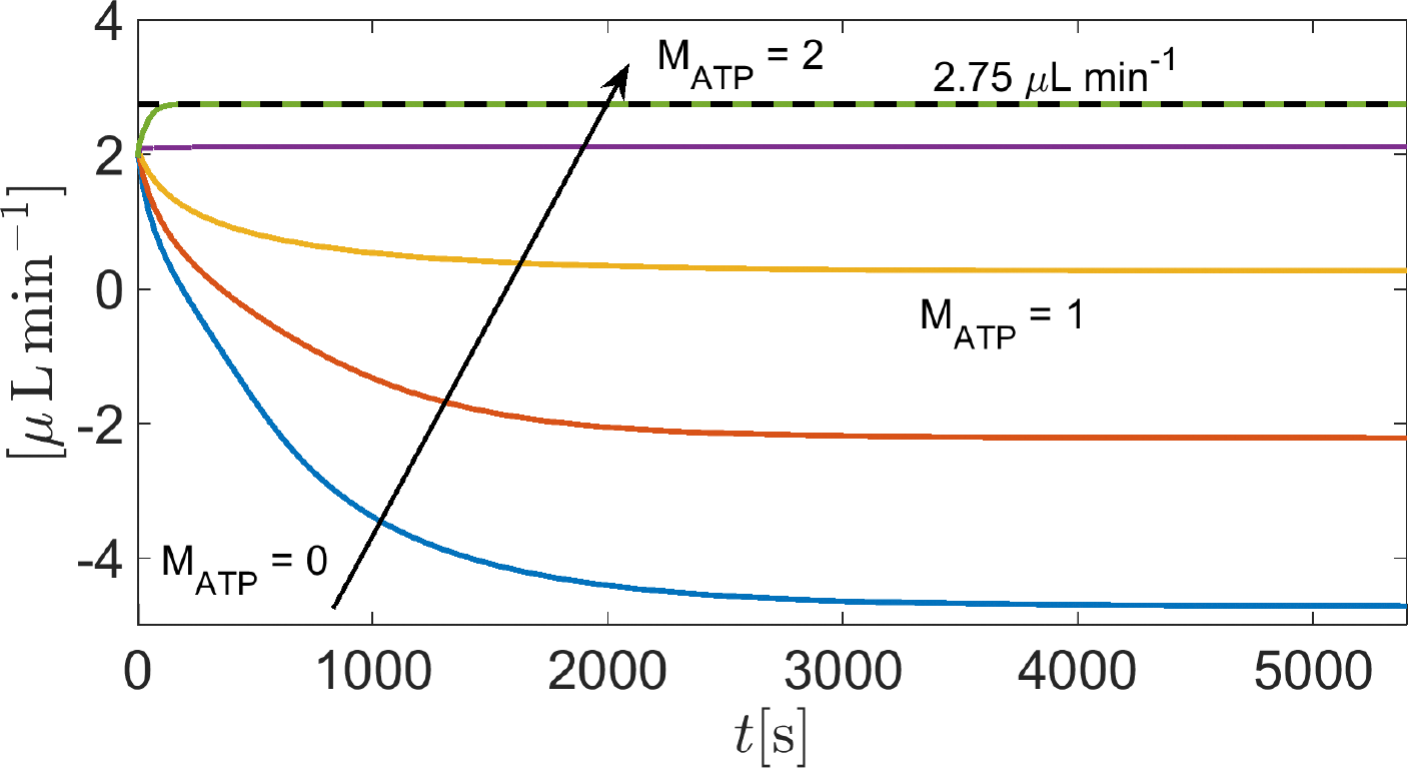
Total AH volumetric flow rate $Q_{AH}$ for $t\in \left[0,\;5400\right]\;s$ as a function of $M_{ATP}$. The black dashed line indicates the physiological value of $Q_{AH}$, equal to 2.75 μL min^−1^, when IOP=15mmHg.

Figure 14 illustrates the time evolution of the total volumetric flow rate $Q_{AH}(t)$ as a function of the time t in the interval $t\in \left[0,\;600\right]\;s$. The results indicate that for $M_{ATP}<1$ (corresponding to values of $c_{ATP}<c_{ATP,\mathrm{ref}}$ ), the predicted total volumetric flow rate of AH is negative. This means that the pump does not have enough energy to move sodium out from the cell and potassium into the cell, so the accumulation of sodium in the cytoplasm attracts chlorine from the extracellular region, eventually leading to the inversion of fluid flow. The predicted total volumetric flow rate of AH becomes positive for $M_{ATP}=1$. This means that the pump does have enough energy to move sodium out from the cell and potassium into the cell, preventing chlorine accumulation in the cell cytoplasm. For increasing values in ATP concentration ( $M_{ATP}>1$ ), water flux is favored, reaching a physiological level when $M_{ATP}=2$.

### 4.3. The Impact of Carbonic Anhydrase

In this simulation, we solve System (38) in the time interval [0, 5400] s with the same set of parameters as in Section 3.2, except the amplification coefficient $A_{CA}$ in Equations (A9c) and (A9d) which is set equal to [0:1:5] (Matlab vector notation). By doing so, the weight of the contribution of the CA enzyme to improve the reaction rate of CO_2_ hydration increases progressively from 0% $\left(A_{CA}=0\right)$ to 100 % $\left(A_{CA}=5\right)$, with respect to the working conditions of Section 3.2, thereby allowing us to investigate the impact of the CA enzyme on the AH simulation. The six computed total AH volumetric flow rates over the considered time interval do not show any visible difference with respect to the change in $A_{CA}$. Therefore, to investigate the impact of CA on AH production, we define the reference AH volumetric flow rate $q_{\mathrm{ref}}=q_{\mathrm{ref}}(t)$ as the function of time predicted by the model for $A_{CA}=0$. Then, we evaluate the maximum percentage variation between each of the other five AH volumetric flow rates predicted by the model and $q_{\mathrm{ref}}$. The results show that the maximum percentage variation ranges between 6.2963 × 10^−3^% and 6.3129 × 10^−3^%. At the same time, the value of the predicted total AH volumetric flow rate for $t\in \left[500,\;5400\right]\;s$ varies between 2.7501731 μL min^−1^ and 2.7501736 μL min^−1^. Correspondingly, the percentage difference between these values and the physiological value of 2.75 μL min^−1^ (for IOP=15mmHg ) ranges between 6.2945 × 10^−3^% and 6.3127 × 10^−3^%. All the above obtained results indicate that $Q_{AH}(t)$ is practically independent of the concentration of the CA enzyme. Further tests with higher values of the amplification parameter $A_{CA}$ do not show significant variation of the predicted value of $Q_{AH}(t)$ ; this probably to be ascribed to the low value of the intracellular carbon dioxide molar density (cf. Figure 11).

### 4.4. The Impact of IOP

In this simulation, we solve System (38) in the time interval [0, 5400] s with the same set of parameters as in Section 3.2, except the value of IOP which is taken in the range [15, 150] mmHg. By doing so, we investigate the response of the model in the case of highly hypertensive patients. In the remainder of this section, we denote by $Q_{AH,b}=2.75\;\mu {Lmin}^{-1}$ the value of the AH volumetric flow rate at the baseline condition IOP=15mmHg and by $Q_{AH}(IOP)$ the value of the AH volumetric flow rate predicted by the model corresponding to a given value of IOP in the range [15, 150] mmHg. We define by

$$\Delta Q_{\%}\left(IOP\right)=\frac{Q_{AH,b}\;-\;Q_{AH}\left(IOP\right)}{Q_{AH,b}}\times 100\;IOP\in \left[15,\;150\right]\;mmHg$$

the percentage difference between the AH volumetric flow rate in baseline conditions and the AH volumetric flow rate predicted by the model corresponding to a given value of IOP in the range [15, 150] mmHg.

**Figure 15.**
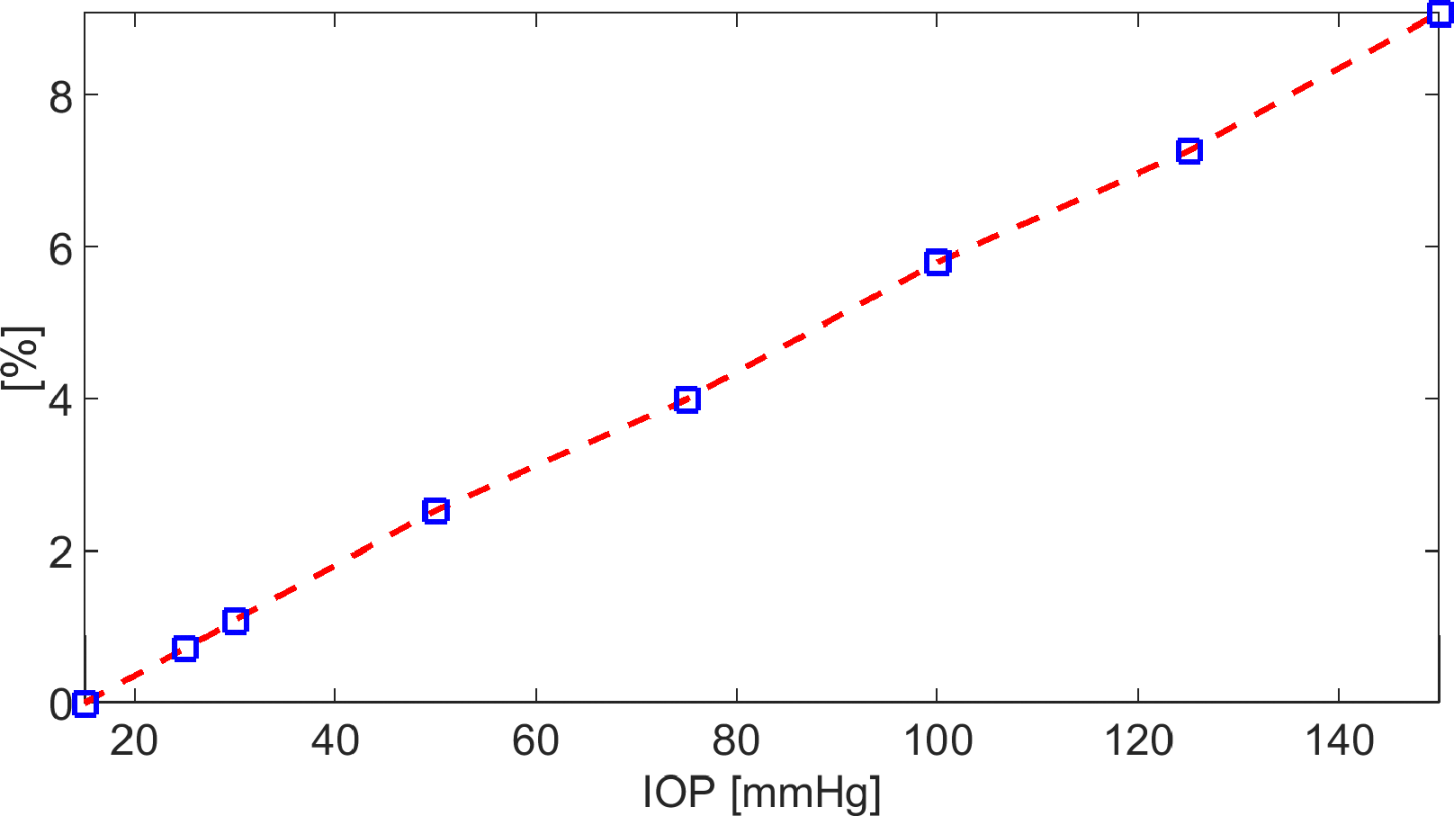
Plot of $\Delta Q_{\%}(IOP)$ for IOP in interval [15, 150] mmHg, with $t\in \left[0,\;5400\right]\;s$.

Figure 15 is the graph of $\Delta Q_{\%}(IOP)$ for IOP in the interval [15, 150] mmHg. The trend is linearly increasing with IOP and indicates that the AH volumetric flow rate predicted by the model decreases with respect to baseline conditions as IOP increases. This agrees with observations in patients affected by Graves’ disease (also known as Thyroid Eye Disease, TED). TED is a chronic, autoimmune, inflammatory orbital disease, causing an increase in the volume and swelling of the soft orbital tissue behind the globe, for which an increase in IOP, as a consequence of an increase in the episcleral vein pressure, determines a reduction in the aqueous humor outflow facility (see [40,41]). The results in Figure 15 also agree with the following expression for fluid velocity:

(48) $$v_{f}=L_{p}\left(p_{\mathrm{in}}(t)-p_{\text{ex}}(t)-\Delta \Pi (t)\right)\;t\geq 0,$$

where $p_{\mathrm{in}}\left(t\right)={\overset{‾}{p}}_{\mathrm{in}}=20\;mmHg$ for t≥0 is the intracellular fluid pressure (corresponding to the estimated fluid pressure in the CE upon assuming 25 mmHg in the ciliary capillaries), $p_{ex}(t)$ is the considered value of IOP in the interval [15, 150] mmHg for t≥0, and ΔΠ(t) is the total osmo-oncotic difference for t≥0. Since ΔΠ(t) turns out to be negative with respect to t, as IOP increases, we see from (48) that $v_{f}$ diminishes, which explains the fact that $Q_{AH}(IOP)$ also diminishes as IOP increases. For IOP=30mmHg, which is a typical value of intraocular pressure in hypertensive individuals (see [42]), we have $\Delta Q_{\%}(30)=1.09\%$, and for IOP=50mmHg, we have $\Delta Q_{\%}(30)=2.54\%$.

## 5. Conclusions

In this article, we proposed, analyzed, and numerically investigated a reduced-order mathematical description of cellular volume homeostasis. The model accounts for intracellular reactions and transmembrane mechanisms for neutral and charged solute exchange. Hydrostatic and osmo-oncotic pressure differences were used in conjunction with Starling’s Law to compute the velocity of the fluid which drives the motion and radial deformation of the cell volume.

The model was implemented within the context of a CVL that was applied to the study of the process of AH production in the human eye. The scope of the simulations was to test the potential of the CVL to assess the relative quantitative importance of the biophysical mechanisms that underlie AH production, from the perspective of their use as supporting tools to integrate and complement in vivo experiments and artificial intelligence-based methodologies for the analysis of data with a statistically significant number of patients.

This study identified for the first time three novel sources of influence on AH production that contribute understanding to previously established models. The first source of influence is that impermeant charged proteins and Na^+^/K^+^ ATPase are important on the level of AH production. The second source of influence is that AH production is independent of CA concentration, at least for low values of CO_2_ concentration. The third source of influence is that AH production decreases with an increase in IOP.

## Figures and Tables

**Table 1.** Model parameters: symbol, value, and units.

Symbol	Value	Units
T	298.15	K
$R_{\mathrm{cell}\;}$	7.816 × 10^−6^	m
$t_{M}$	7.5 × 10^−9^	m
$c_{M}$	5.9 × 10^−3^	F m^−2^
${\overset{‾}{p}}_{in}$	20	mmHg
${\overset{‾}{p}}_{ex}$	15	mmHg
$\Delta \overset{‾}{p}$	5	mmHg
$\sigma_{\beta }$	[0.1, 0.1]^*T*^	[·]
$\sigma_{\alpha }$	[0.3, 0.3, 0.3, 0.3, 0.3]^*T*^	[·]
$\sigma_{X}$	1	[·]
$P_{\beta }$	[0.228, 0.1467]^*T*^	m s^−1^
$P_{\alpha }$	[0.0013, 0.2613, 1.1587, 0.5227, 0.1467]^*T*^	m s^−1^
$\Phi_{\beta }^{\mathrm{carr}\;}$	10^−3^[0.1352, 0.1352]^*T*^	[·]
$\Phi_{\alpha }^{\mathrm{ch}}$	10^−4^ [0.0126, 0.0785, 0.0031, 0.0196, 0.1539]^*T*^	[·]
$\Phi^{AQP}$	1.3515 × 10^−4^	[·]
$\Phi_{\alpha }^{\mathrm{pump}\;}$	[0.0011, 0.0011, 0, 0, 0]^*T*^	[·]
$\Phi^{\mathrm{lip}}$	0.9974	[·]

**Figure A1.**
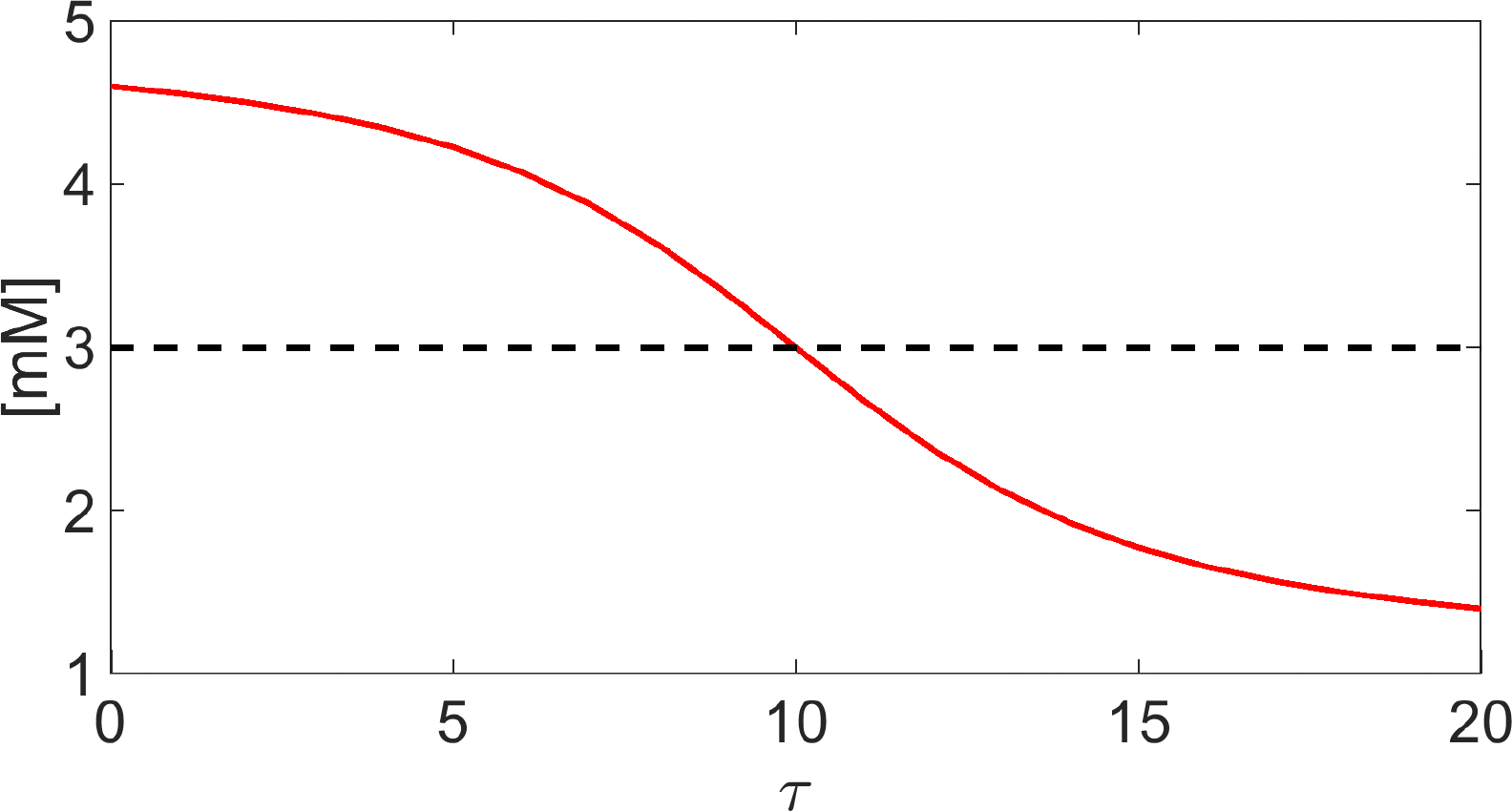
Red solid line: a plot of $\left\langle c_{\alpha }\right\rangle$ for τ=[0:20] and $X_{\alpha }(\tau )=\tau -10$, with τ being a dimensionless time. The endpoint values of $c_{\alpha }$ are $c_{\alpha ,in}\left(\tau \right)=1\;mM$ and $c_{\alpha ,ex}\left(t\right)=5\;mM$ for every τ∈[0:20]. Black dashed line: the arithmetic average of $c_{\alpha }$.

**Figure A2.**
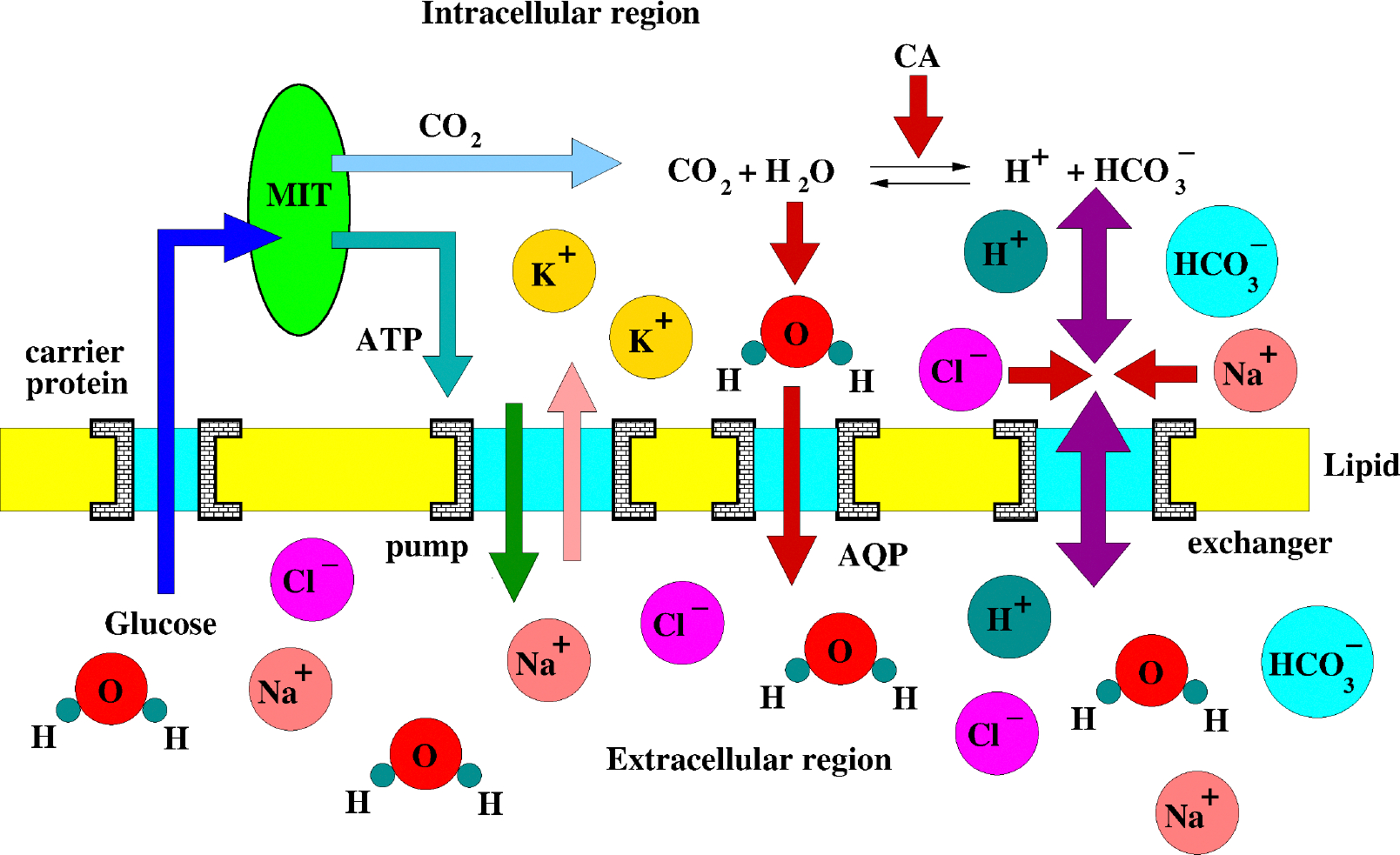
Schematic representation of intracellular reactions and transmembrane transport mechanisms. MIT: mitochondrium. ATP: adenosinetriphosphate. CA: carbonic anhydrase. Exchangers perform multiple ion transport across membrane.

## Data Availability

The original contributions presented in this study are included in the article. Further inquiries can be directed to the corresponding author.

## Appendix A. Normal Fluid Velocity and Solute Molar Flux Densities

In this section, we provide the expressions of the normal fluid velocity on the cell surface and solute molar flux densities in (36).

### Appendix A.1. Normal Fluid Velocity Inside the Pore Channel of the AQP

Let us proceed by proving Equation (11a). The expression of the fluid stress tensor in cylindrical coordinates is

(A1a)
$$T=\left[\begin{array}{ccc} -p(r,s,\theta ,t) & 0 & \mu_{f}\frac{\partial V_{s}(r,t)}{\partial r}\\ 0 & -p(r,s,\theta ,t) & 0\\ \mu_{f}\frac{\partial V_{s}(r,t)}{\partial r} & 0 & -p(r,s,\theta ,t) \end{array}\right],$$

so its divergence is the following vector:

(A1b)
$$\nabla \cdot T=\left[\begin{array}{c} \frac{1}{r}\frac{\partial }{\partial r}(-rp(r,s,\theta ,t))\\ \frac{1}{r}\frac{\partial }{\partial \theta }(-p(r,s,\theta ,t))\\ \frac{1}{r}\frac{\partial }{\partial r}\left(r\mu_{f}\frac{\partial V_{s}(r,t)}{\partial r}\right)-\frac{\partial p(r,s,\theta ,t)}{\partial s} \end{array}\right].$$

The linear momentum Equation (5b) becomes

(A1c)
$$\frac{1}{r}\frac{\partial }{\partial r}(-rp(r,s,\theta ,t))=0,$$

(A1d)
$$\frac{1}{r}\frac{\partial }{\partial \theta }(-p(r,s,\theta ,t))=0,$$

(A1e)
$$\frac{1}{r}\frac{\partial }{\partial r}\left(r\mu_{f}\frac{\partial V_{s}(r,\;t)}{\partial r}\right)-\frac{\partial p(r,\;s,\;\theta ,\;t)}{\partial s}+b_{p,s}(s,t)=0.$$

Equations (A1c) and (A1d) imply that p does not depend on r and θ, i.e., p=p(s,t). Equation (A1e) becomes

(A1f)
$$\frac{1}{r}\mu_{f}v_{p,n}(t)\frac{\partial }{\partial r}\left(r\frac{d\eta (r)}{dr}\right)-\frac{\partial p(s,\;t)}{\partial s}+b_{p,s}(s,t)=0.$$

Replacing the expression of η in (A1f) yields

$$\frac{1}{r}\mu_{f}v_{p,n}(t)\frac{\partial }{\partial r}\left(-\frac{4r^{2}}{r_{p}^{2}}\right)-\frac{\partial p(s,\;t)}{\partial s}+b_{p,s}(s,t)=0,$$

from which we obtain

$$-\frac{8\mu_{f}}{r_{p}^{2}}v_{p,n}(t)-\frac{\partial p(s,\;t)}{\partial s}+b_{p,s}(s,t)=0.$$

Rearranging the above equation, we obtain the following expression for the normal fluid velocity in the AQP:

$$v_{p,n}(t)=\frac{r_{p}^{2}}{8\mu_{f}}\left[-\frac{\partial p(s,\;t)}{\partial s}+b_{p,s}(s,t)\right],$$

which is Equation (11a).

### Appendix A.2. Normal Fluid Velocity on the Cell Surface

To determine the normal fluid velocity on the cell surface, we consider the following two cases:

**(B.C.1)** The hydraulic pressure difference $\Delta \overset{‾}{p}(t)$ is given (units: Pa).

**(B.C.2)** The total water volumetric flow rate across the cell surface ${\overset{‾}{Q}}_{w,cell}(t)$ is given (units: m^3^ s^−1^).

In case (B.C.2), we recall the definition of the water volumetric flow rate across the cell surface:

(A2)
$$Q_{w,cell}(t)=\int_{S_{t}}v_{fl}(x,t)\cdot nd\left(S_{t}\right)\;t\geq 0.$$

Since $Q_{w,cell}(t)$ is given and equal to ${\overset{‾}{Q}}_{w,cell}(t)$, we obtain

(A3)
$$v_{cell,n}(t)=\frac{{\overset{‾}{Q}}_{w,cell}(t)}{𝒮(t)}=\frac{{\overset{‾}{Q}}_{w,cell}(t)}{\gamma (𝒱(t){)}^{2/3}}\;t\geq 0,$$

where we use the fact that $𝒮(t)=\gamma (𝒱(t){)}^{2/3}$.

In case (B.C.1), we use (11d) to obtain

(A4)
$$v_{cell,n}(t)=\Phi^{AQP}v_{p,n}(t)=\Phi^{AQP}{ℒ}_{p}[\Delta \overset{‾}{p}(t)-\Delta \Pi (t)]\;t\geq 0.$$

To determine $v_{cell,n}$, we need to provide a mathematical model for the total osmo-oncotic pressure difference ΔΠ. Let us define the electrochemical potential of the ion α :

(A5a)
$$\varphi_{\alpha }^{ec}=\psi +\frac{V_{th}}{z_{\alpha }}\ln\left(\frac{c_{\alpha }}{c_{ref}}\right)\;\alpha \in S_{\alpha },$$

where $c_{ref}$ is a reference molar density. Let us introduce the pressure differences:

(A5b)
$$\Delta \Pi_{\alpha }(t):=\int_{0}^{t_{m}}-\sigma_{\alpha }Fz_{\alpha }c_{\alpha }(s,\;t)\frac{\partial \varphi_{\alpha }^{ec}(s,\;t)}{\partial s}ds\;\alpha \in S_{\alpha },\;t\geq 0,$$

(A5c)
$$\Delta \Pi_{\beta }(t):=\int_{0}^{t_{m}}-\sigma_{\beta }RT\frac{\partial c_{\beta }(s,\;t)}{\partial s}ds\;\beta \in S_{\beta }\;t\geq 0,$$

(A5d)
$$\Delta \Pi_{X}(t):=\int_{0}^{t_{m}}-\sigma_{X}RT\frac{\partial c_{X}(s,\;t)}{\partial s}ds\;t\geq 0.$$

The quantities $\Delta \Pi_{\alpha }$ and $\Delta \Pi_{\beta }$ are the osmotic pressure differences associated with the charged solute $\alpha \in S_{\alpha }$ and neutral solute $\beta \in S_{\beta }$, respectively, whereas $\Delta \Pi_{X}$ is the oncotic pressure difference associated with the impermeant charge $c_{X}$. The quantity R is the gas constant (units: J mol^−1^ K^−1^), and $\sigma_{\alpha }$, $\sigma_{\beta }$, and $\sigma_{X}$ are the membrane reflection coefficients associated with the ion α, solute β, and impermeant fixed charge $c_{X}$, respectively. We notice that each coefficient is in the range [0, 1], with the value 0 corresponding to a completely permeable membrane and the value 1 corresponding to a completely impermeable membrane. To evaluate the integral on the right-hand side of (A5b), we replace $c_{\alpha }(s,t)$ with its spatial average:

(A5e)
$$\left\langle c_{\alpha }\right\rangle (t):=\frac{\int_{0}^{t_{m}}c_{\alpha }(s,\;t)\;ds}{t_{m}}\;t\geq 0.$$

Postponing to Appendix A.4 the computation of (A5e) and replacing it in (A5b), we obtain

(A5f)
$$\Delta \Pi_{\alpha }(t)=\sigma_{\alpha }Fz_{\alpha }\left\langle c_{\alpha }\right\rangle (t)\Delta \varphi_{\alpha }^{ec}(t)\;\alpha \in S_{\alpha }\;t\geq 0,$$

(A5g)
$$\Delta \Pi_{\beta }(t)=\sigma_{\beta }RT\Delta c_{\beta }(t)\;\beta \in S_{\beta }\;t\geq 0,$$

(A5h)
$$\Delta \Pi_{X}(t)=\sigma_{X}RT\Delta c_{X}(t)\;t\geq 0.$$

We define the total osmotic pressure difference as

(A5i)
$$\Delta \Pi_{osm}(t)=\sum_{\alpha \in S_{\alpha }}\Delta \Pi_{\alpha }(t)+\sum_{\beta \in S_{\beta }}\Delta \Pi_{\beta }(t)\;t\geq 0,$$

and the oncotic pressure difference as

(A5j)
$$\Delta \Pi_{onc}(t)=\Delta \Pi_{X}(t)\;t\geq 0.$$

Then, we define the total osmo-oncotic pressure difference as

(A5k)
$$\begin{array}{l} \Delta \Pi (t)=\Delta \Pi_{osm}(t)+\Delta \Pi_{onc}(t)\\ \;=\sum_{\alpha \in S_{\alpha }}\sigma_{\alpha }\left[Fz_{\alpha }\left\langle c_{\alpha }\right\rangle (t)\psi_{m}(t)+RT\left\langle c_{\alpha }\right\rangle (t)\;\ln\left(\frac{c_{\alpha ,in\;}(t)}{c_{\alpha ,ex}(t)}\right)\right]\\ \;+\;RT\sum_{\beta \in S_{\beta }}\sigma_{\beta }\Delta c_{\beta }(t)+\sigma_{X}RT\Delta c_{X}(t)\;t\geq 0. \end{array}$$

Relation (A5k) is the generalization of Equation (31) in [4].

### Appendix A.3. Neutral Solutes

Let us consider a carrier protein whose geometrical structure is the same as that of the pore $\omega_{p}$, illustrated in Figure 5, in the case of an AQP. To determine the normal molar flux density of the solute β inside $\omega_{p}$, we make the following assumptions.

#### Assumption A1.

*We assume the following:*

1. *The molar flux density of the neutral solute* β *only has the axial component* $j_{\beta ,s}$;
2. *For any* t≥0, *the quantity* $j_{\beta ,s}$ *the quantity* $\omega_{p}$, *i.e.*, $j_{\beta ,s}=j_{\beta ,n}(t)$, *with* t≥0 *and* $s\in \left[0,t_{m}\right]$.

Using Assumption A1, Assumption 4-A3, and the fact that $v_{p,n}$ is spatially constant in the constitutive Equation (6b) gives the following second-order equation for the solute molar density inside the carrier protein channel:

(A6a)
$$v_{p,n}(t)\frac{\partial c_{\beta }(s,\;t)}{\partial s}-D_{\beta }\frac{\partial^{2}c_{\beta }(s,\;t)}{\partial s^{2}}=0\;t\geq 0,s\in \left[0,t_{m}\right],$$

whose solution is

(A6b)
$$c_{\beta }(s,\;t)=A(t)+B(t)\;\exp\left(\frac{v_{p,n}(t)}{D_{\beta }}s\right)\;t\geq 0,s\in \left[0,t_{m}\right],$$

with A=A(t) and B=B(t) being time-dependent arbitrary functions. Inserting (A6b) into the constitutive Equation (6b) yields

(A6c)
$$j_{\beta ,n}(t)=v_{p,n}(t)A(t)\;t\geq 0.$$

To determine A(t), we need to enforce the following boundary conditions:

(A6d)
$$c_{\beta }(0,t)=c_{\beta ,in}(t),\;c_{\beta }\left(t_{m},t\right)=c_{\beta ,ex}(t)\;t\geq 0,$$

where $c_{\beta ,in}(t)$ and $c_{\beta ,ex}(t)$ are the intra- and extracellular values of the neutral solute molar density for any t≥0. Using (A6d) in (A6b), we obtain the following expression of the normal molar flux density of β inside the carrier protein channel:

(A6e)
$$j_{\beta ,n}(t)=-P_{\beta }\left[c_{\beta ,ex}(t)\mathrm{Be}\left(X_{\beta }(t)\right)-c_{\beta ,in}(t)\mathrm{Be}\left(-X_{\beta }(t)\right)\right]\;t\geq 0,$$

where $P_{\beta }:=D_{\beta }/t_{m}$ is the membrane permeability of the neutral solute β (units: ms^−1^),

$$\mathrm{Be}\left(W\right)≔\frac{W}{e^{W}\;-\;1}\;W\in R$$

is the inverse of the Bernoulli function, and

(A6f)
$$X_{\beta }(t):=\frac{v_{p,n}(t)}{P_{\beta }}\;t\geq 0.$$

### Appendix A.4. Charged Solutes

We can proceed in the same way as in Appendix A.3 to obtain the following expression for the normal molar flux density of the ion $\alpha \in S_{\alpha }$ inside an ionic channel.

(A7a)
$$j_{\alpha ,n}^{edw}(t)=-P_{\alpha }\left[c_{\alpha ,ex}(t)Be\left(X_{\alpha }(t)\right)-c_{\alpha ,in}(t)Be\left(-X_{\alpha }(t)\right)\right]\;t\geq 0,$$

where $P_{\alpha }:=D_{\alpha }/t_{m}$ is the membrane permeability of the charged solute α and

(A7b)
$$X_{\alpha }(t):=\frac{v_{p,n}(t)}{P_{\alpha }}+z_{\alpha }\frac{\psi_{m}(t)}{V_{th\;}}\;t\geq 0.$$

Using the definition (8e) of the generalized drift velocity of the ion α in (A7b), we find the spatial distribution of $c_{\alpha }$ inside the ionic channel:

(A7c)
$$c_{\alpha }(s,\;t)=\tilde{A}(t)+\tilde{B}(t)\;\exp\left(\frac{v_{\alpha ,n}(t)}{D_{\alpha }}s\right)\;t\geq 0,s\in \left[0,t_{m}\right],$$

where $\tilde{A}=\tilde{A}(t)$ and $\tilde{B}=\tilde{B}(t)$ are time-dependent arbitrary functions, and $v_{\alpha ,n}(t)$ is the average normal component of $v_{\alpha }=v_{\alpha }(x,t)$ on the cell surface, with $x\in \partial \Omega_{t}$ and t≥0. Replacing (A7c) in (A5e), we obtain

(A7d)
$$\left\langle c_{\alpha }\right\rangle (t)=c_{\alpha ,in}(t)\xi_{in}(t)+c_{\alpha ,ex}(t)\xi_{ex}(t)\;t\geq 0,$$

where

(A7e)
$$\xi_{in\;}(t)=\frac{Be\left(-X_{\alpha }(t)\right)\;-\;1}{X_{\alpha }(t)}\;t\geq 0.$$

(A7f)
$$\xi_{ex}(t)=\frac{1\;-\;Be\left(X_{\alpha }(t)\right)}{X_{\alpha }(t)}\;t\geq 0.$$

We illustrate in Figure A1 a graphical representation of $\left\langle c_{\alpha }\right\rangle (\tau )$ (red solid line) compared to the arithmetic value $c_{\alpha }^{arith}(\tau )=\left(c_{\alpha ,in}(\tau )+c_{\alpha ,ex}(\tau )\right)/2$ for τ=[0:20] (Matlab vector notation); $X_{\alpha }(\tau )=\tau -10,c_{\alpha ,in}(\tau )=1mM$ ; and $c_{\alpha ,ex}\left(\tau \right)=5\;mM$. We see that $\left\langle c_{\alpha }\right\rangle$ and $c_{\alpha }^{arith}$ are comparably close only if $\left|X_{\alpha }\right|≃0$, which corresponds to a diffusive regime of transport inside the channel. Conversely, their distance increases for larger values of $\left|X_{\alpha }\right|$, which corresponds to an advection-dominated regime of transport inside the channel. On the basis of these considerations, the adoption of (A7d) instead of the customary choice $c_{\alpha }^{arith}$ (as performed, for example, in [4]) is expected to warrant an accurate and robust model simulation.

## Appendix B. Mathematical Modeling of Cellular Metabolism

The following conceptual scheme of cellular metabolism is considered in this article:

1. Glucose is absorbed by mitochondria to produce ATP and CO_2_.
2. ATP provides the energy needed by the Na^+^/K^+^ pump to export three sodium ions and import two potassium ions.
3. The carbonic anhydrase enzyme (CA) catalizes the hydrolysis of CO_2_, which is a waste product of mitochondrial metabolism.
4. Specialized exchangers supervise the transmembrane transport of the proton (H^+^) and bicarbonate (${\text{HCO}}_{3}^{-}$), which are the products of CO_2_ hydrolysis.

Figure A2 illustrates the intracellular reactions and transmembrane transport mechanisms that are involved in cellular metabolism.

In the following, we provide the expressions of:

1. The production and consumption rates for the neutral and charged solutes involved in the CA enzyme-mediated carbon dioxide conversion into carbonic acid and its subsequent dissociation into protonated hydrogen and bicarbonate;
2. The production and consumption rates in cellular volume regulation;
3. The molar flux densities representing the mathematical model of transmembrane sodium and potassium solute exchange throughout Na^+^/K^+^ ATPase.

### Appendix B.1. Mathematical Model of CA-Mediated CO2 Hydrolysis

In this section, we illustrate the mathematical modeling of the CA enzyme-mediated CO_2_ hydrolysis. This process can be conveniently represented as the following two-step chemical reaction (see [43,44]):

(A8a)
$${\mathrm{STEP}\;1:CO}_{2}+H_{2}O⇄H_{2}{CO}_{3}.$$

(A8b)
$$\mathrm{STEP}\;2:H_{2}{CO}_{3}⇄H^{+}+{HCO}_{3}^{-}.$$

STEP 1 is the conversion of intracellular carbon dioxide into carbonic acid under the mediation of the CA enzyme, which is a very fast catalyzer of the CO_2_ hydration process (see [45,46]). STEP 1 is characterized by the quantities $k_{hydr}$ and $k_{dehydr}$ (units: s^−1^) depending on the molar density of the carbonic anhydrase enzyme (CA) and representing the rate constants of the forward and backward reactions in STEP 1, respectively.

#### Assumption A2.

*Let* $c_{{CO}_{2(aQ),in}}$ *denote the molar density of intracellular CO*_2_ *that is hydrated by water molecules. We assume that each CO*_2_ *molecule that is produced by mitochondria’s respiration is hydrated by a corresponding water molecule. This allows us to denote by* $c_{{CO}_{2,in}}$ *the molar density of the intracellular* $c_{{CO}_{2(aQ)}}$.

Using the Law of Conservation of Mass and Assumption A2, the net production rates in the mass balance equation for $\beta ={CO}_{2}$ and $\beta =H_{2}{CO}_{3}$ have the following expressions:

(A9a)
$${ℛ}_{{CO}_{2}}(t)=k_{dehydr}c_{H_{2}{CO}_{3,\;in}(t)}-k_{hydr}c_{{CO}_{2,\;in}}(t)\;t\geq 0,$$

(A9b)
$${ℛ}_{H_{2}{CO}_{3}}\left(t\right)=-{ℛ}_{{CO}_{2}}\left(t\right)\;t\geq 0.$$

The hydration and dehydration rate constants experimentally depend on the amount of CA that is present in the compartment where the hydration reaction takes place (see [47]). Following [43,44], in this article, we use the following model for the hydration and dehydration rate constants:

(A9c)
$$k_{hydr}=A_{CA}k_{hydr,ref},$$

(A9d)
$$k_{dehydr}=A_{CA}k_{dehydr,ref},$$

where $A_{CA}$ is a nonnegative given constant, whereas $k_{hydr,ref\;}=0.037{\;s}^{-1}$ and $k_{dehydr,ref\;}=\;13.7{\;s}^{-1}$ are experimental reference values measured at $t=25^{\circ }C$, reported in [48]. Taking $A_{CA}>1$ is a way to represent the catalyzing effect of CA compared to the uncatalyzed reaction corresponding to $A_{CA}=1$. In the numerical simulations illustrated in Section 3.2, we set $A_{CA}=5$.

STEP 2 is the dissociation of intracellular carbonic acid into bicarbonate and protonated hydrogen and is characterized by the quantities $k_{diss}$ (units: mM^−1^ s^−1^) and $k_{assoc}$ (units: mM^−1^ s^−1^), the rate constants of the forward and backward reactions in STEP 2, respectively. The dissociation reaction of H_2_CO_3_ is extremely rapid, so the values of the forward and backward rate constants in STEP 2 are very large. In the numerical simulations illustrated in Section 3.2, we use the data of [43,44] and set $k_{diss\;}=10^{16}{\;s}^{-1}$ and $k_{assoc}=k_{diss}/K_{eQ}$, where $K_{eQ}=0.2804\;mM$ is the equilibrium constant of STEP 2. Using the Law of Conservation of Mass, we obtain the following expressions for the net production rates in the mass balance equations for $\alpha =H^{+}$ and $\alpha ={HCO}_{3}^{-}$:

(A10)
$${ℛ}_{\alpha }(t)=k_{diss}c_{H_{2}{CO}_{3}}(t)-k_{assoc}c_{H^{+}}(t)c_{{HCO}_{3}^{-}}(t)\;\alpha =\left\{H^{+},{HCO}_{3}^{-}\right\},\;t\geq 0.$$

#### Assumption A3.

*We assume that the ions* $\alpha \in \left\{{Na}^{+},K^{+},{Cl}^{-}\right\}$ *are non-reacting (see* [49] *(Section 8.3.3)). Therefore, we have* ${ℛ}_{\alpha }(t)=0$, *with* t≥0.

### Appendix B.2. Net Production Rate in Cell Volume Regulation

The mechanisms which govern intracellular water production/consumption are the object of considerable debate and investigation because of their importance in cell life and survival. One example is provided by the process of normotonic cell shrinkage which is the major hallmark of cellular apoptosis [50]. Another example is provided by the TCA cycle (Krebs cycle) in cellular respiration, whose products of metabolism of fuels are ATP, CO_2_, and so-called “metabolic” water [51].

The difficulty of accessing and sampling the contents of intact cells makes the study of the intracellular fluid environment, and, more generally, of body fluid content a challenging problem. Specific approaches to accurately detect metabolic water content as a result of intracellular metabolic activity have been proposed in [52,53].

In this article, we focus on the role of the net production and consumption of intracellular water, $R_{w}$, in driving the motion of the cell surface. Our proposed model is

(A11)
$${ℛ}_{w}(t)=k_{w,p}-k_{w,c}\frac{𝒱(t)}{{𝒱}_{0}}\;t\geq 0,$$

where $k_{w,p}$ and $k_{w,c}$ are given constants (units: s^−1^) and ${𝒱}_{0}$ is the value of cell volume in resting conditions. In the simulations illustrated in Section 3.2, we set $k_{w,p}=k_{w,c}=1{\;s}^{-1}$.

### Appendix B.3. Na^+^/K^+^ ATPase

The mathematical model of the sodium–potassium pump (Na^+^/K^+^ ATPase) adopted in this article follows the idea proposed in [3]. The molar flux densities for sodium and potassium are

(A12a)
$$j_{Na,n}^{a}(t)=j_{pump}(t)\;t\geq 0,$$

(A12b)
$$j_{K,n}^{a}(t)=-\frac{2}{3}j_{pump}(t)\;t\geq 0,$$

where

(A12c)
$$j_{pump}(t)=j_{pump\;}^{MAX\;}{\left(\frac{c_{{Na}_{in}}(t)}{c_{{Na}_{in}}(t)\;+\;c_{Na,\;1/2}}\right)}^{3}{\left(\frac{c_{K_{ex}}(t)}{c_{K_{ex}}(t)\;+\;c_{K,\;1/2}}\right)}^{2}\;t\geq 0,$$

(A12d)
$$j_{pump\;}^{MAX}:=c_{ATP}v_{pump}.$$

The quantity $j_{pump}^{MAX}$ is the maximum pump molar flux density (units: mM ms^−1^), $c_{ATP\;}$ is the intracellular molar density of ATP (units: mM), and $v_{pump}$ is the ion transfer velocity of the pump (units: ms^−1^). The quantities $c_{ATP}$ and $v_{pump}$ are defined as

(A12e)
$$c_{ATP}=M_{ATP}c_{ATP,ref},$$

(A12f)
$$v_{pump}=r_{turn}t_{M},$$

where $c_{ATP,ref}$ is the reference value of the ATP molar density (units: mM), $r_{turn}$ is pump turnover rate (units: s^−1^), and $M_{ATP}$ is a nonnegative given constant. The quantities $c_{Na,1/2}$ and $c_{K,1/2}$ are the Michaelis constants of the pump model (units: mM). In the simulations illustrated in Section 3.2, we set $c_{ATP,ref\;}=10^{-2}\;mM$, $M_{ATP}=2$, $r_{turn\;}=10^{7}{\;s}^{-1}$, $c_{Na,1/2}=1.3\;mM$ and $c_{K,1/2}=0.14\;mM$.

## Abbreviations

The following abbreviations are used in this manuscript:

AH: Aqueous humor
CA: Carbonic anhydrase
ATP: Adenosinetriphosphate
IOP: Intraocular pressure
CVL: Computational virtual laboratory
AQP: Aquaporin
BE: Backward Euler
CN: Crank–Nicolson
CE: Ciliary epithelium
